# Supplementary figures and images for: The giant staphylococcal protein Embp facilitates colonization of surfaces through Velcro-like attachment to fibrillated fibronectin
Source: eLife. 2022 Jul 7;11:e76164. doi: 10.7554/eLife.76164 (PMC9302970; doi:10.7554/eLife.76164)

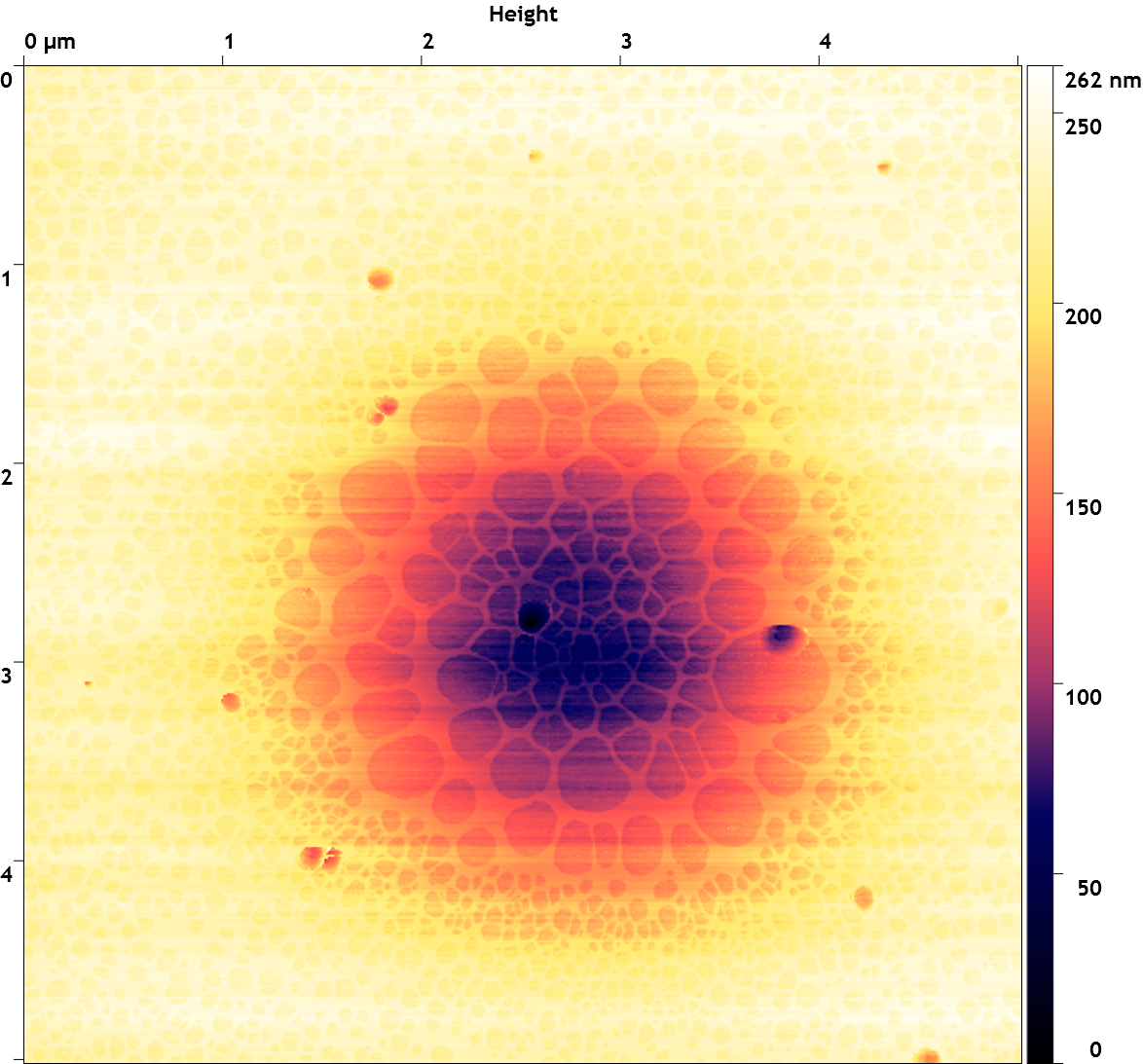

Supplement: Figure 3—source data 1. [file elife-76164-fig3-data1.zip › Figure 3 source data/Figure 3A source data/qi-fit-2018.01.07-22.29.50.004.png]

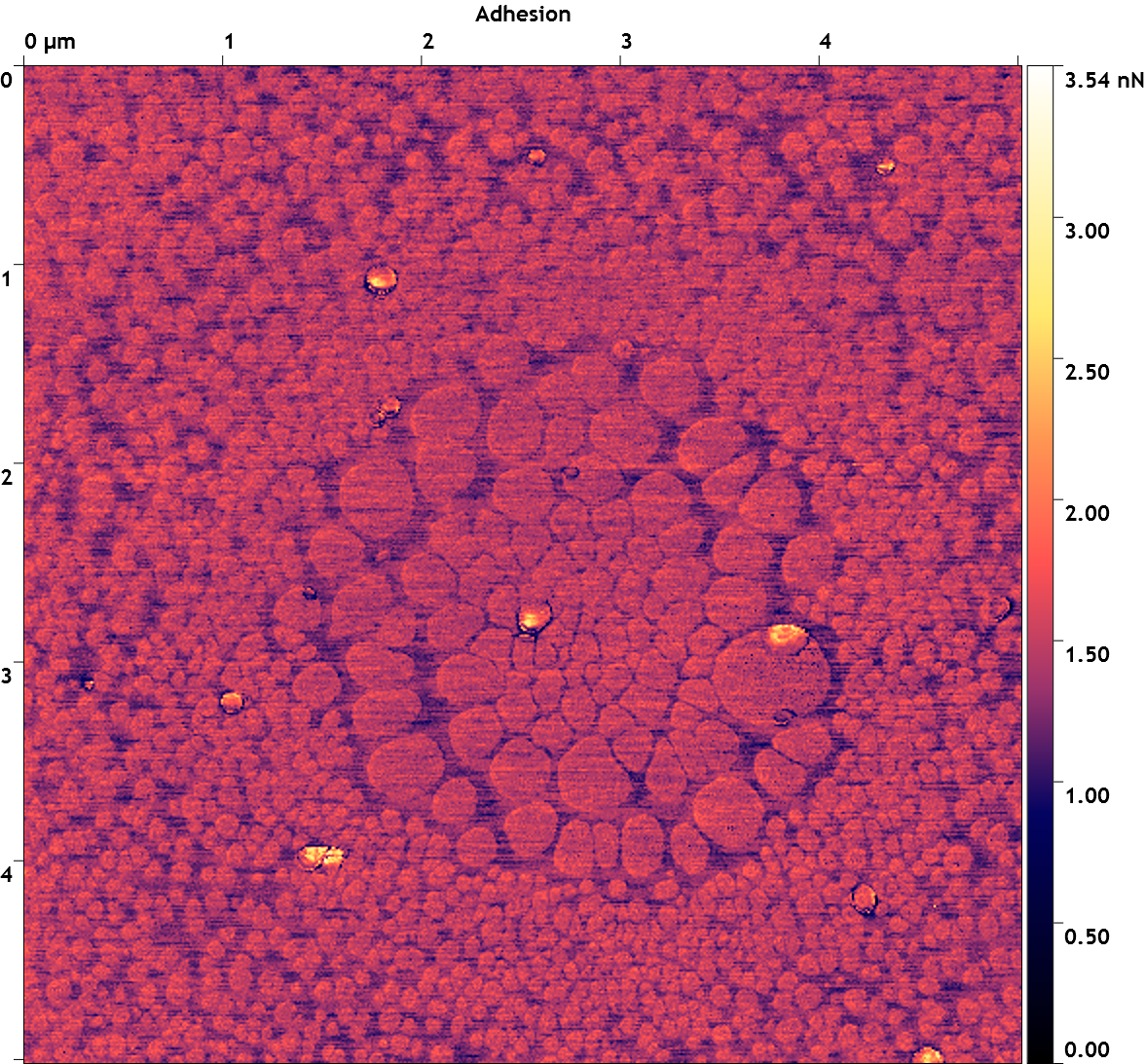

Supplement: Figure 3—source data 1. [file elife-76164-fig3-data1.zip › Figure 3 source data/Figure 3A source data/qi-fit-2018.01.07-22.29.50.004ad.png]

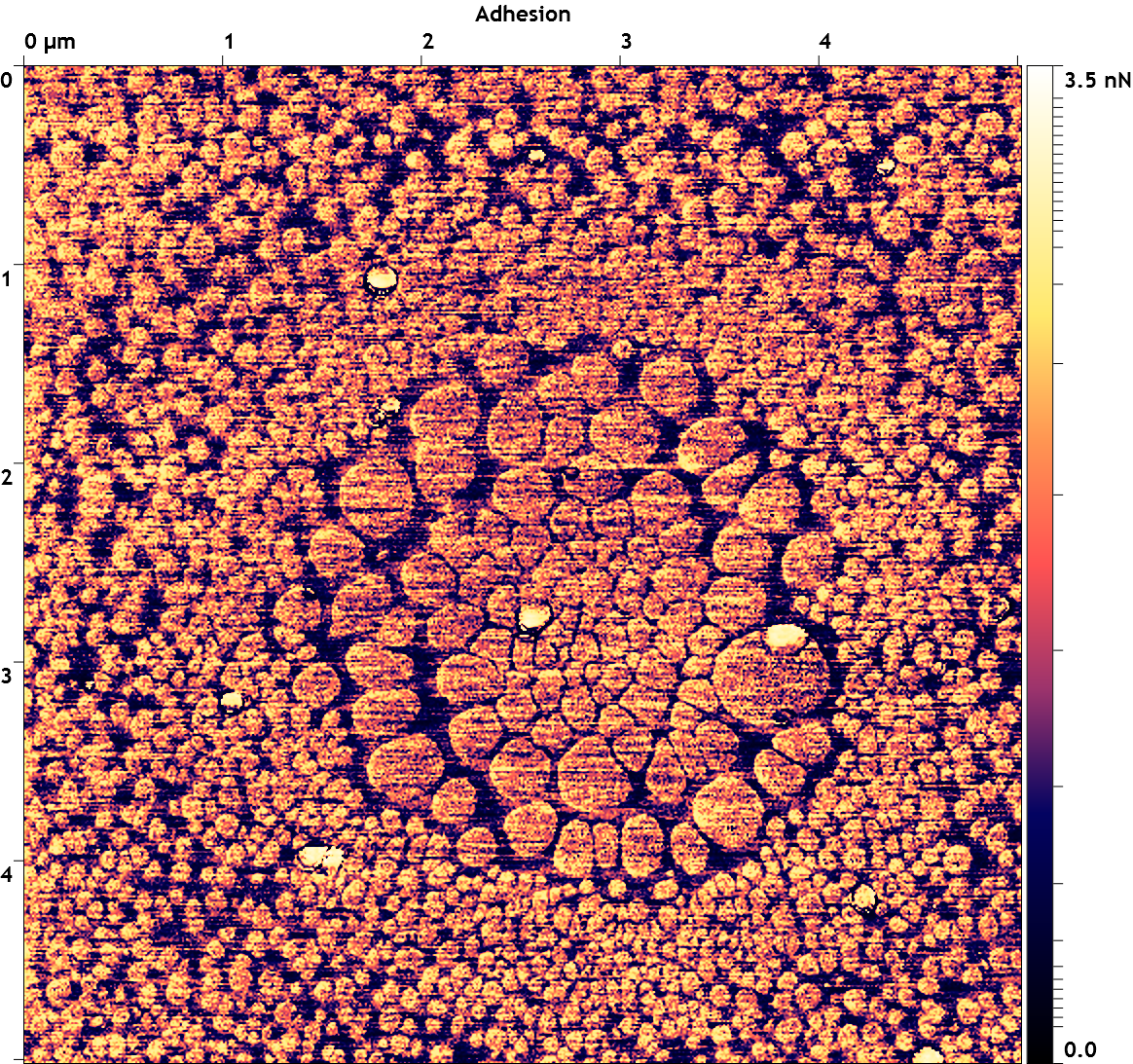

Supplement: Figure 3—source data 1. [file elife-76164-fig3-data1.zip › Figure 3 source data/Figure 3A source data/qi-fit-2018.01.07-22.29.50.004adb.png]

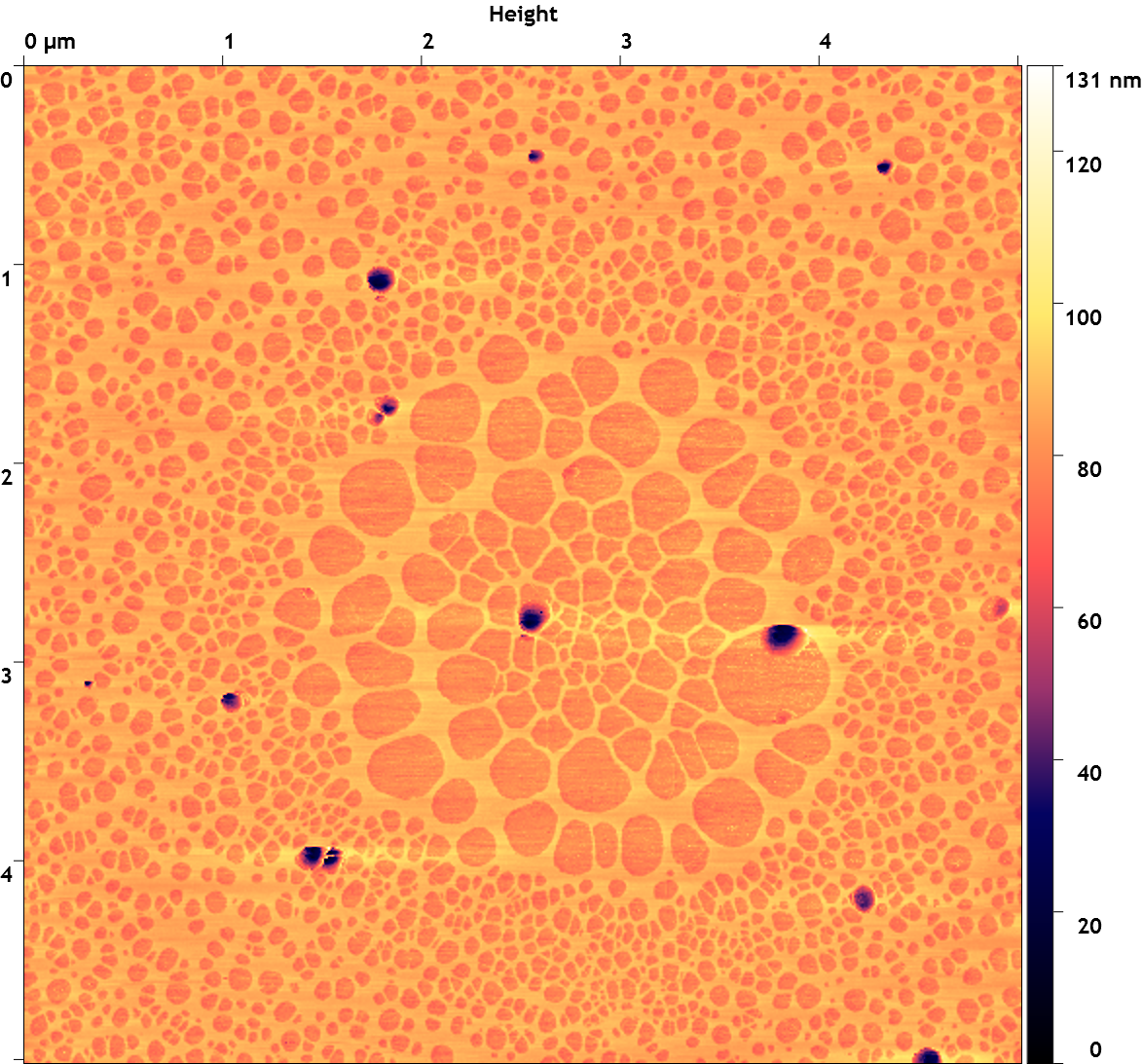

Supplement: Figure 3—source data 1. [file elife-76164-fig3-data1.zip › Figure 3 source data/Figure 3A source data/qi-fit-2018.01.07-22.29.50.004f.png]

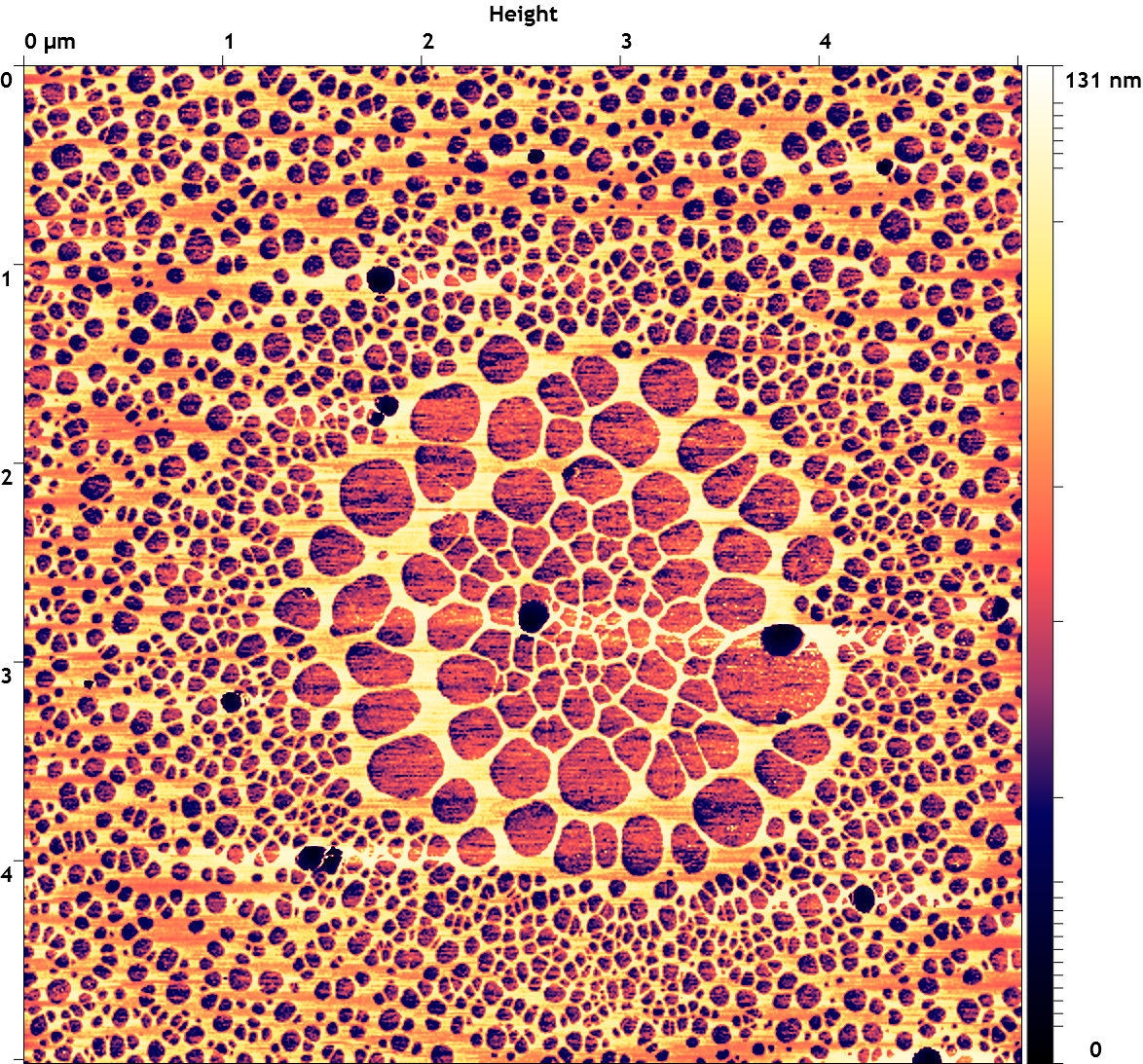

Supplement: Figure 3—source data 1. [file elife-76164-fig3-data1.zip › Figure 3 source data/Figure 3A source data/qi-fit-2018.01.07-22.29.50.004fb.png]

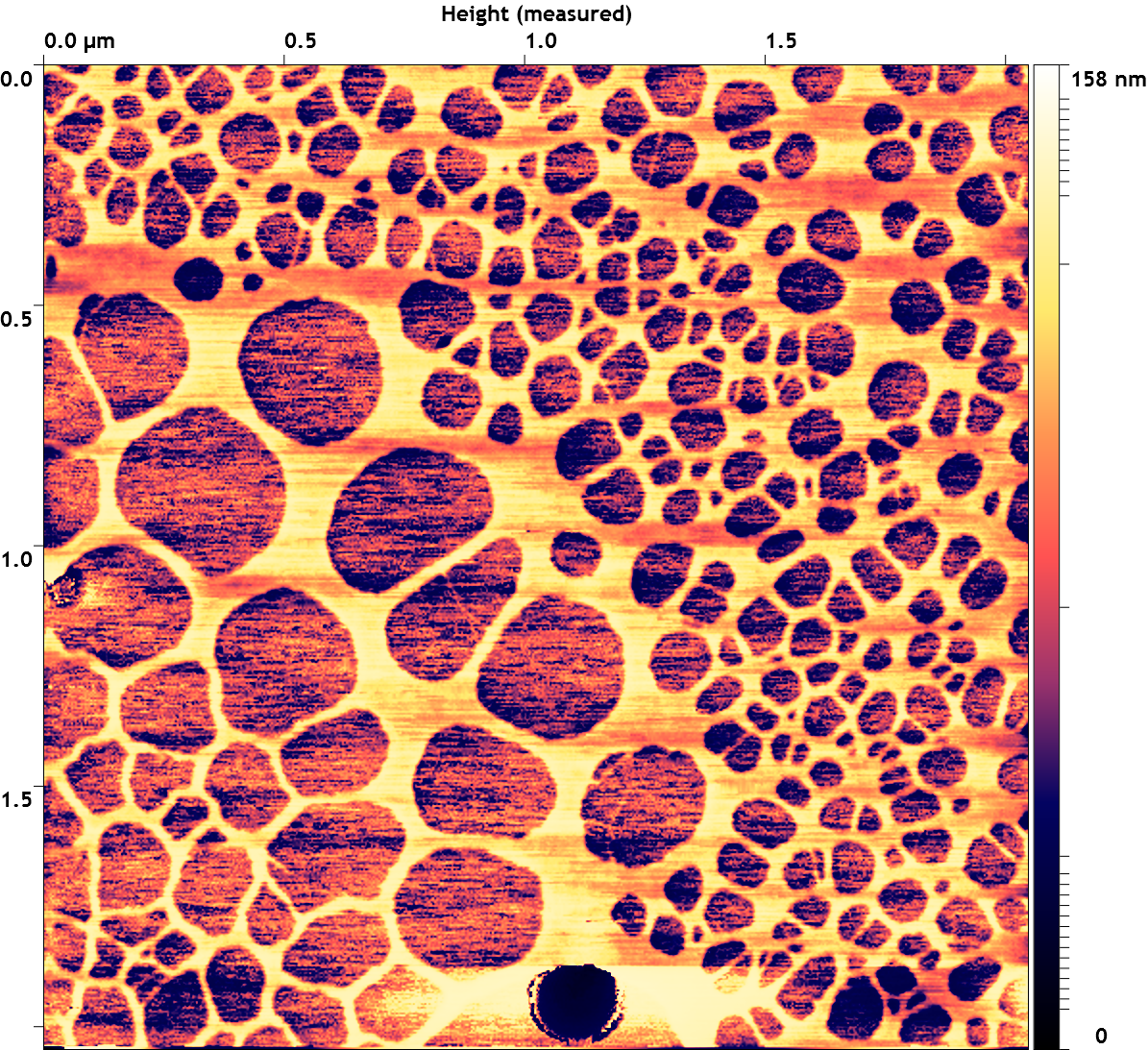

Supplement: Figure 3—source data 1. [file elife-76164-fig3-data1.zip › Figure 3 source data/Figure 3A source data/qi-fit-2018.01.07-22.58.08.855.png]

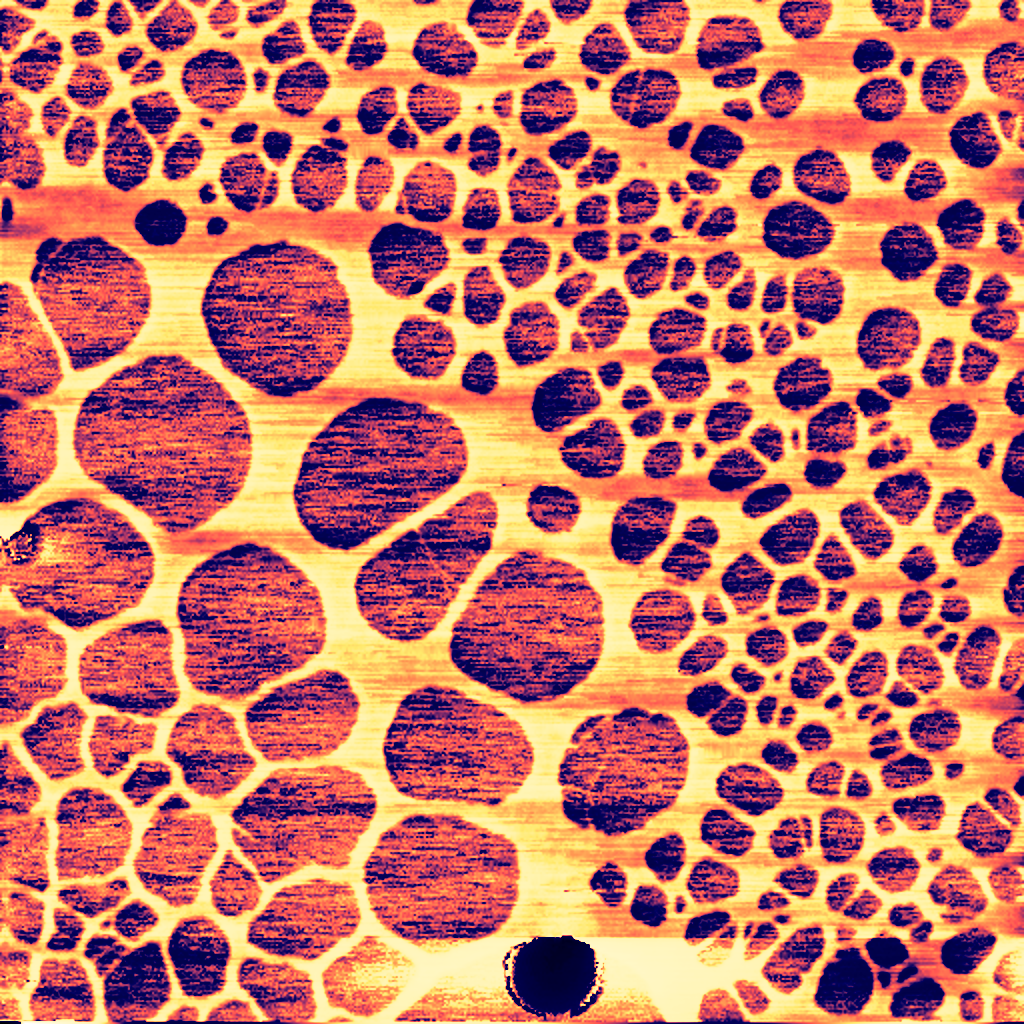

Supplement: Figure 3—source data 1. [file elife-76164-fig3-data1.zip › Figure 3 source data/Figure 3A source data/qi-fit-2018.01.07-22.58.08.855CUT.png]

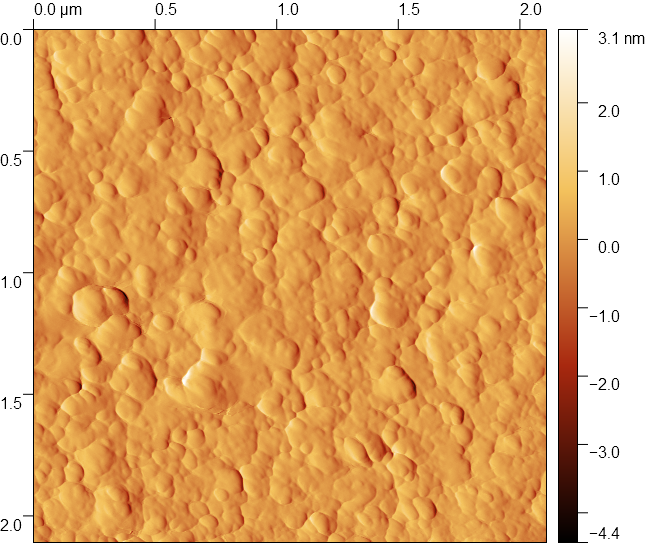

Supplement: Figure 3—source data 1. [file elife-76164-fig3-data1.zip › Figure 3 source data/Figure 3B source data/20min 0.35mg-ml 5u diffuse 40ul wash 1time 20200605.0_00080_AmplitudeErr.png]

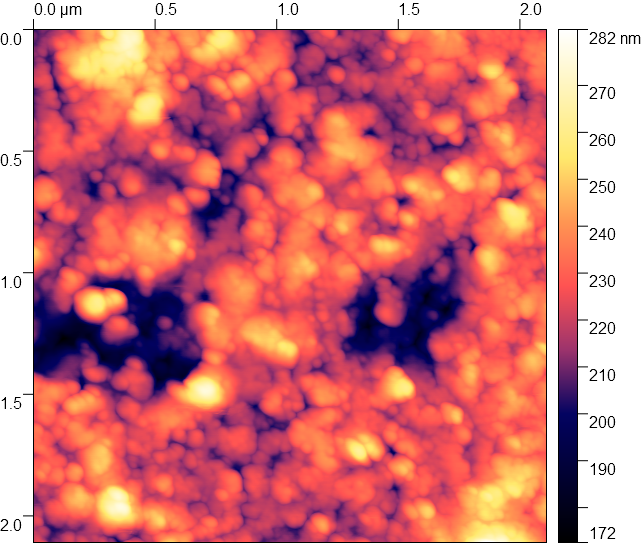

Supplement: Figure 3—source data 1. [file elife-76164-fig3-data1.zip › Figure 3 source data/Figure 3B source data/20min 0.35mg-ml 5u diffuse 40ul wash 1time 20200605.0_00080_Height_RT.png]

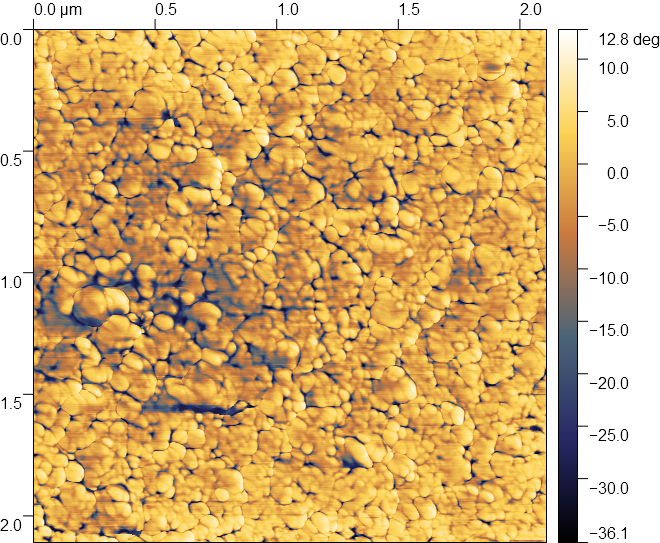

Supplement: Figure 3—source data 1. [file elife-76164-fig3-data1.zip › Figure 3 source data/Figure 3B source data/20min 0.35mg-ml 5u diffuse 40ul wash 1time 20200605.0_00080_Phase.png]

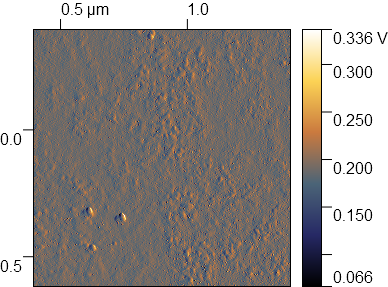

Supplement: Figure 3—figure supplement 1—source data 1. [file elife-76164-fig3-figsupp1-data1.zip › Figure 3 suppl 1 source data/Clean_glass-2017.12.28-18.41.50.912_AmpErr.png]

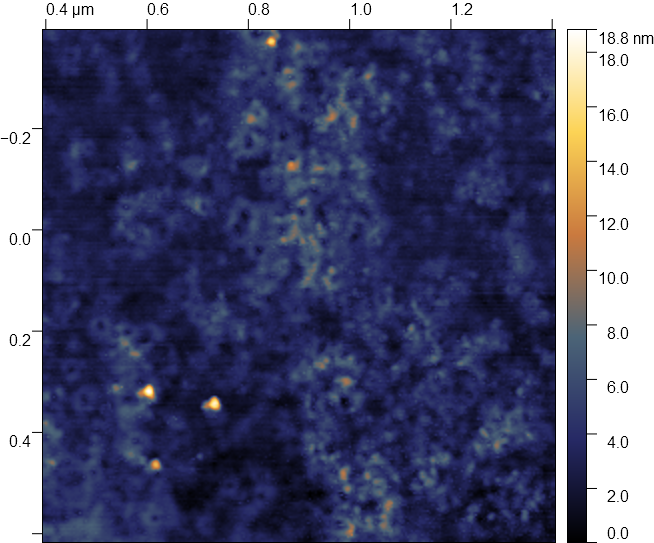

Supplement: Figure 3—figure supplement 1—source data 1. [file elife-76164-fig3-figsupp1-data1.zip › Figure 3 suppl 1 source data/Clean_glass-2017.12.28-18.41.50.912_Height.png]

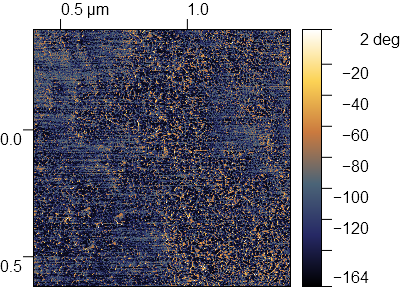

Supplement: Figure 3—figure supplement 1—source data 1. [file elife-76164-fig3-figsupp1-data1.zip › Figure 3 suppl 1 source data/Clean_glass-2017.12.28-18.41.50.912_Phase.png]

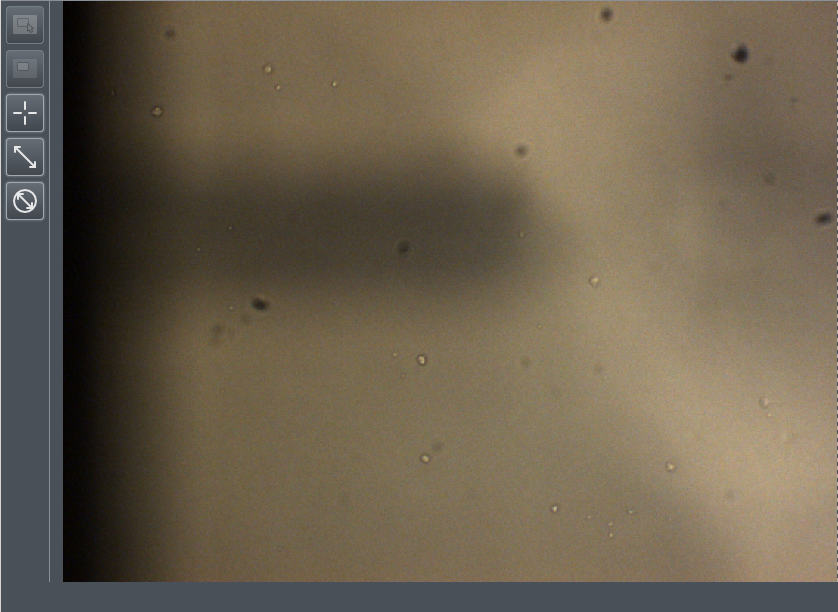

Supplement: Figure 5—source data 1. [file elife-76164-fig5-data1.zip › Figure 5 source data/Cell pickup 1.png]

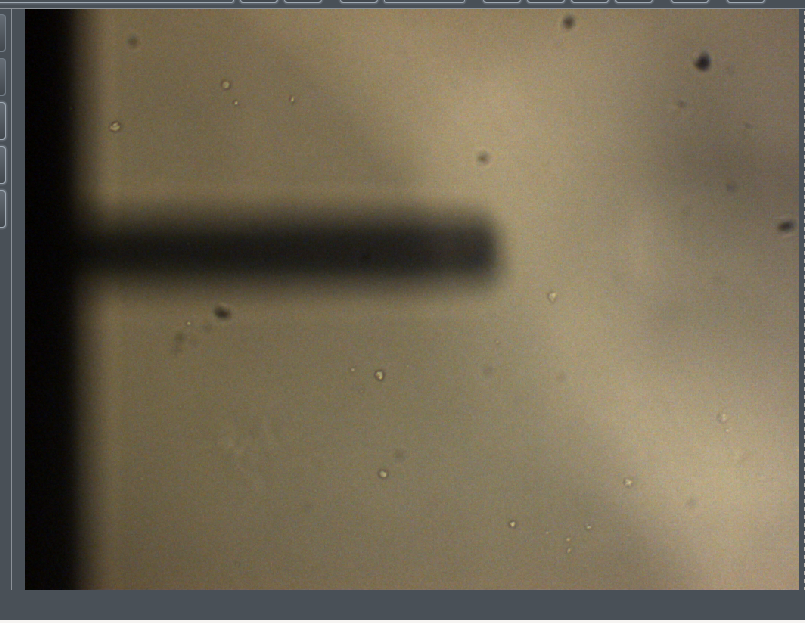

Supplement: Figure 5—source data 1. [file elife-76164-fig5-data1.zip › Figure 5 source data/Cell pickup 2.png]

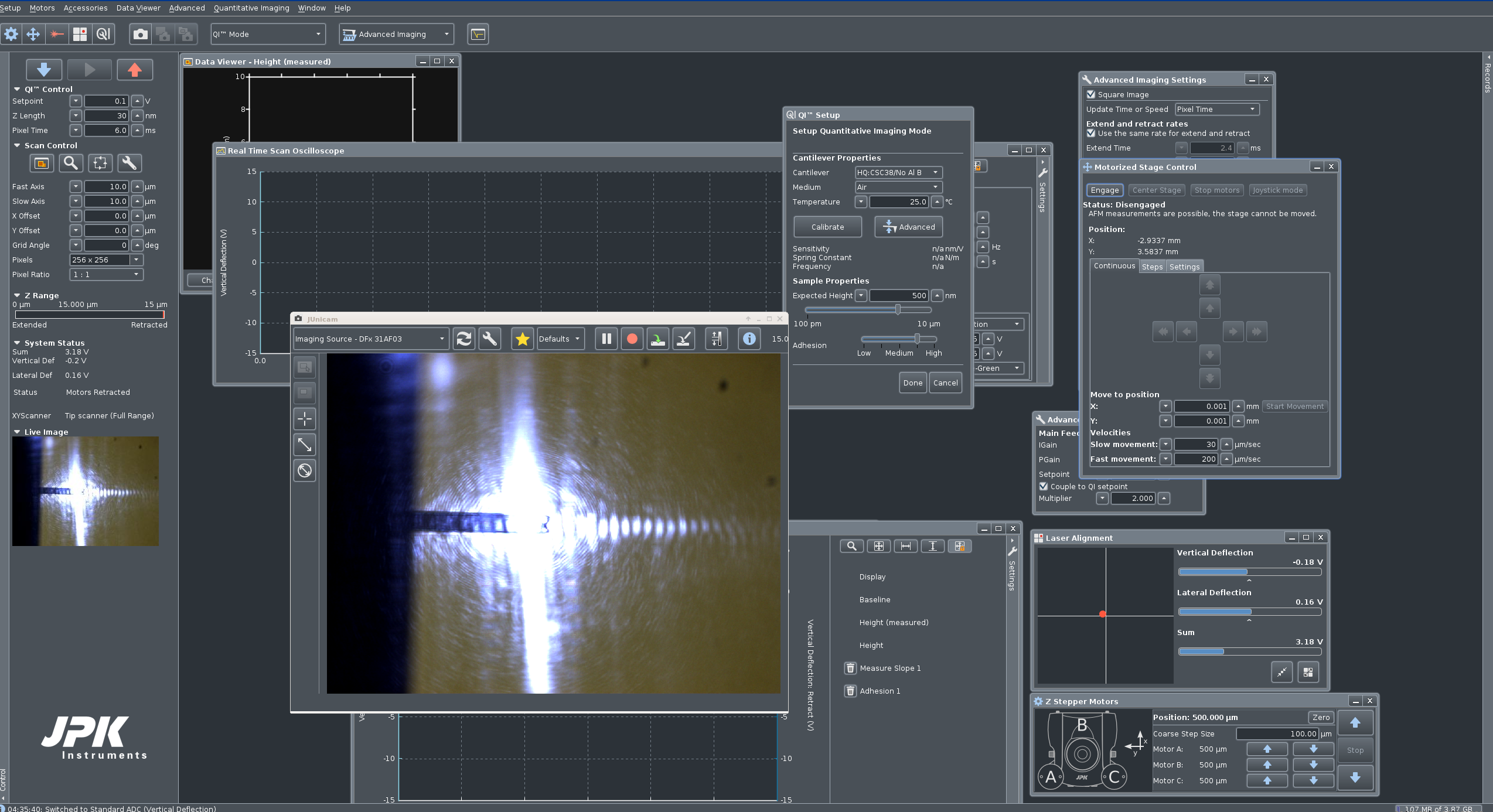

Supplement: Figure 5—source data 1. [file elife-76164-fig5-data1.zip › Figure 5 source data/System overview/Screenshot - 01.png]

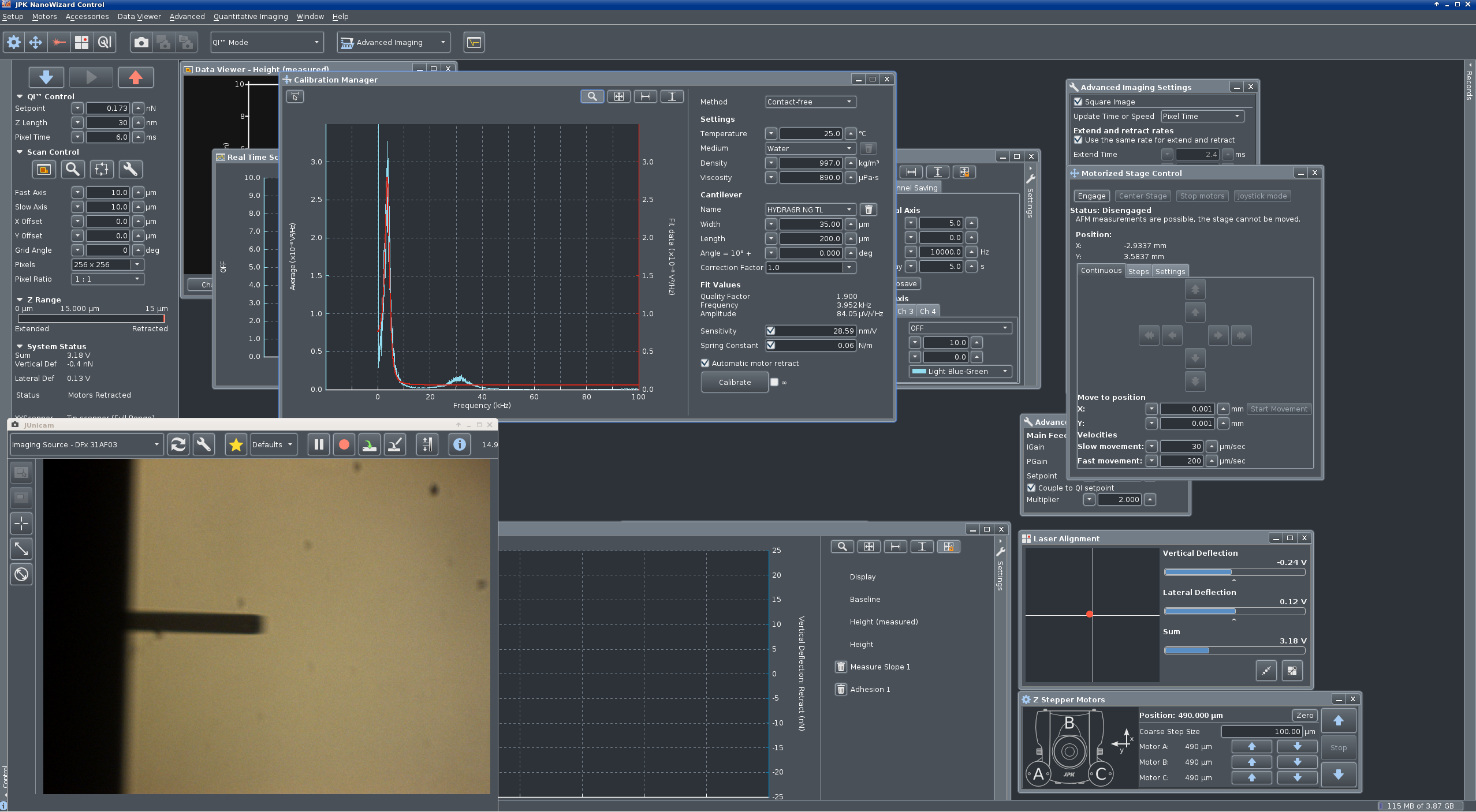

Supplement: Figure 5—source data 1. [file elife-76164-fig5-data1.zip › Figure 5 source data/System overview/Screenshot - 02.png]

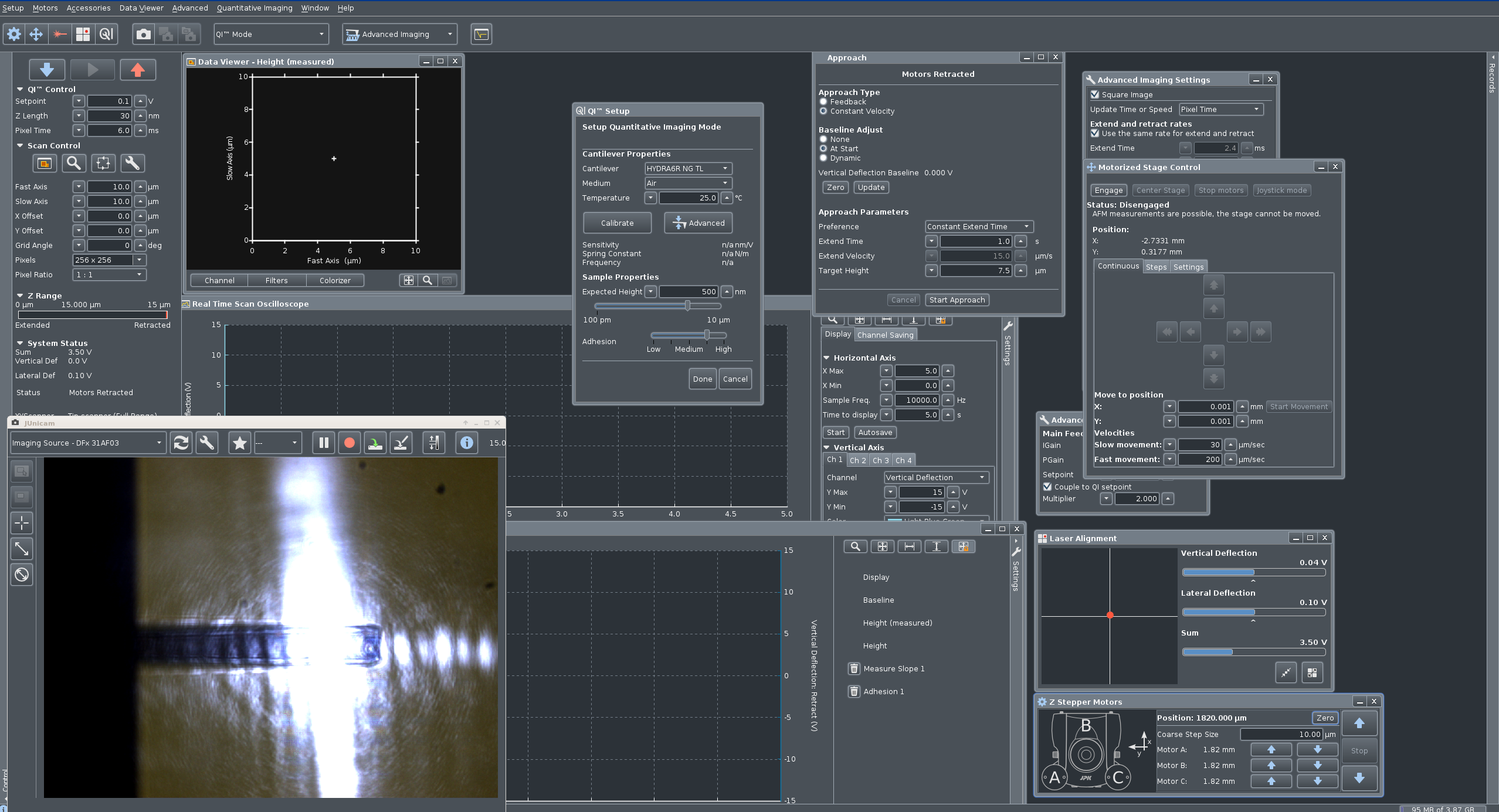

Supplement: Figure 5—source data 1. [file elife-76164-fig5-data1.zip › Figure 5 source data/System overview/Screenshot - 03.png]

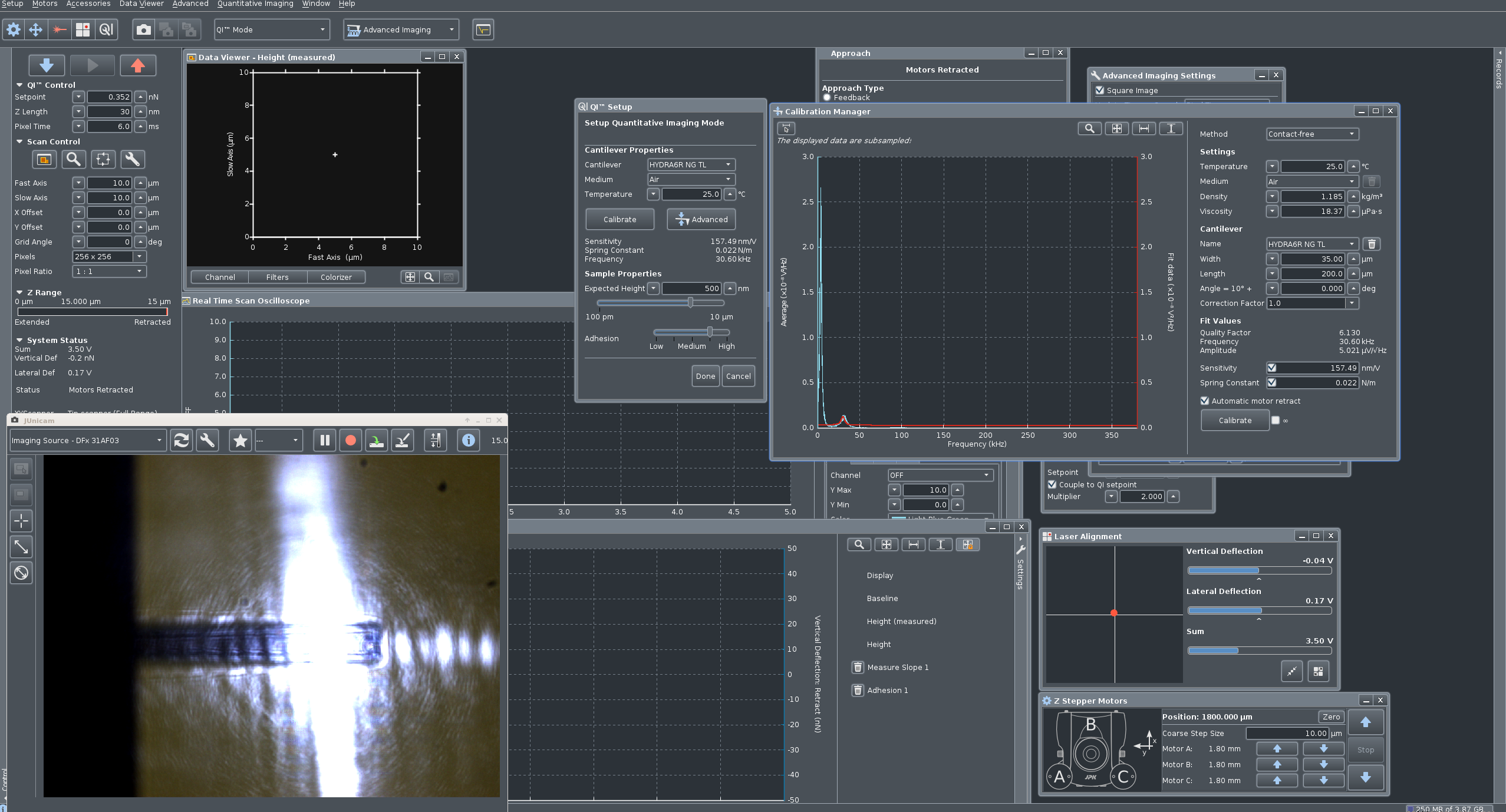

Supplement: Figure 5—source data 1. [file elife-76164-fig5-data1.zip › Figure 5 source data/System overview/Screenshot - 04.png]

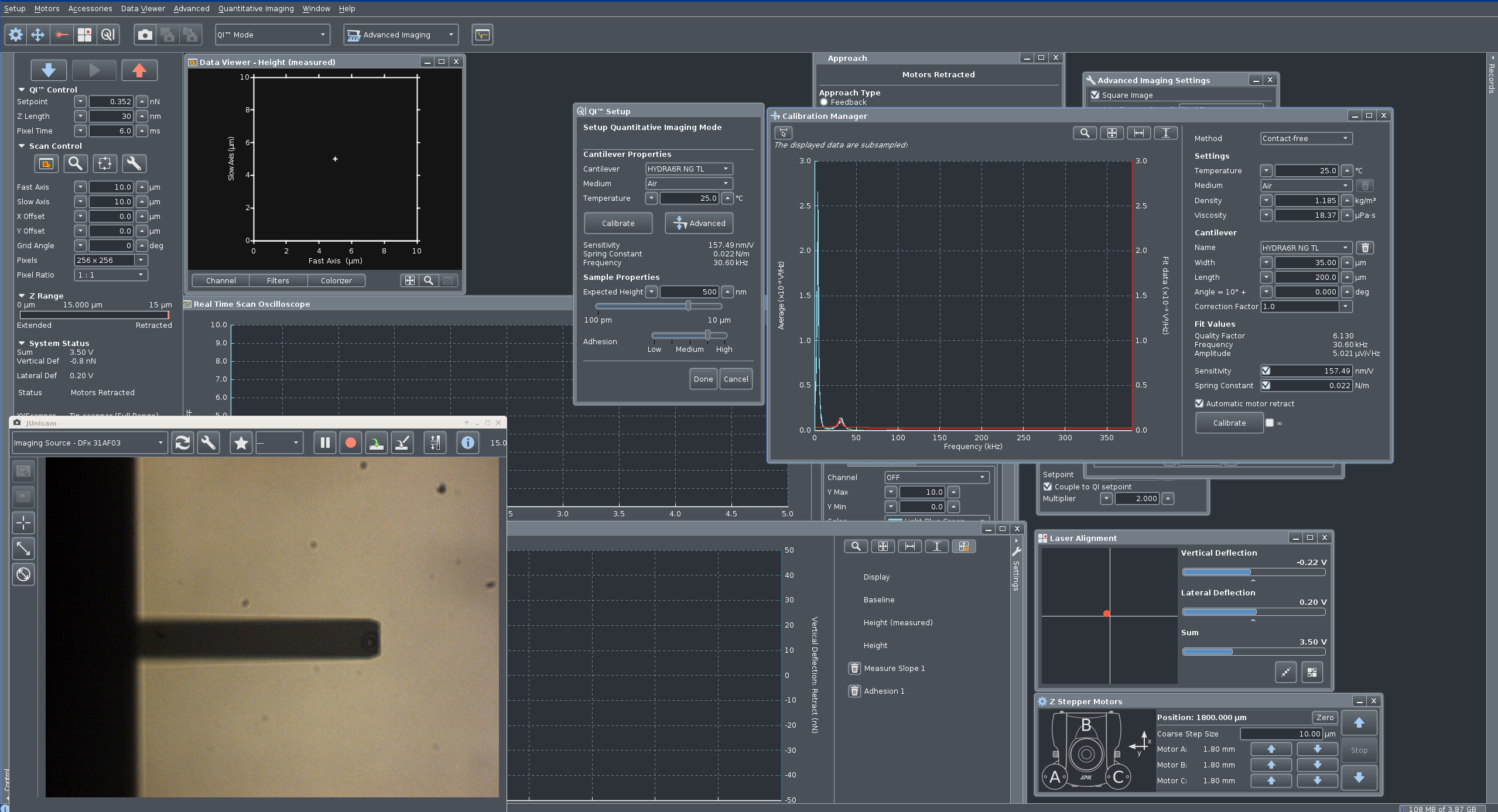

Supplement: Figure 5—source data 1. [file elife-76164-fig5-data1.zip › Figure 5 source data/System overview/Screenshot - 05.png]

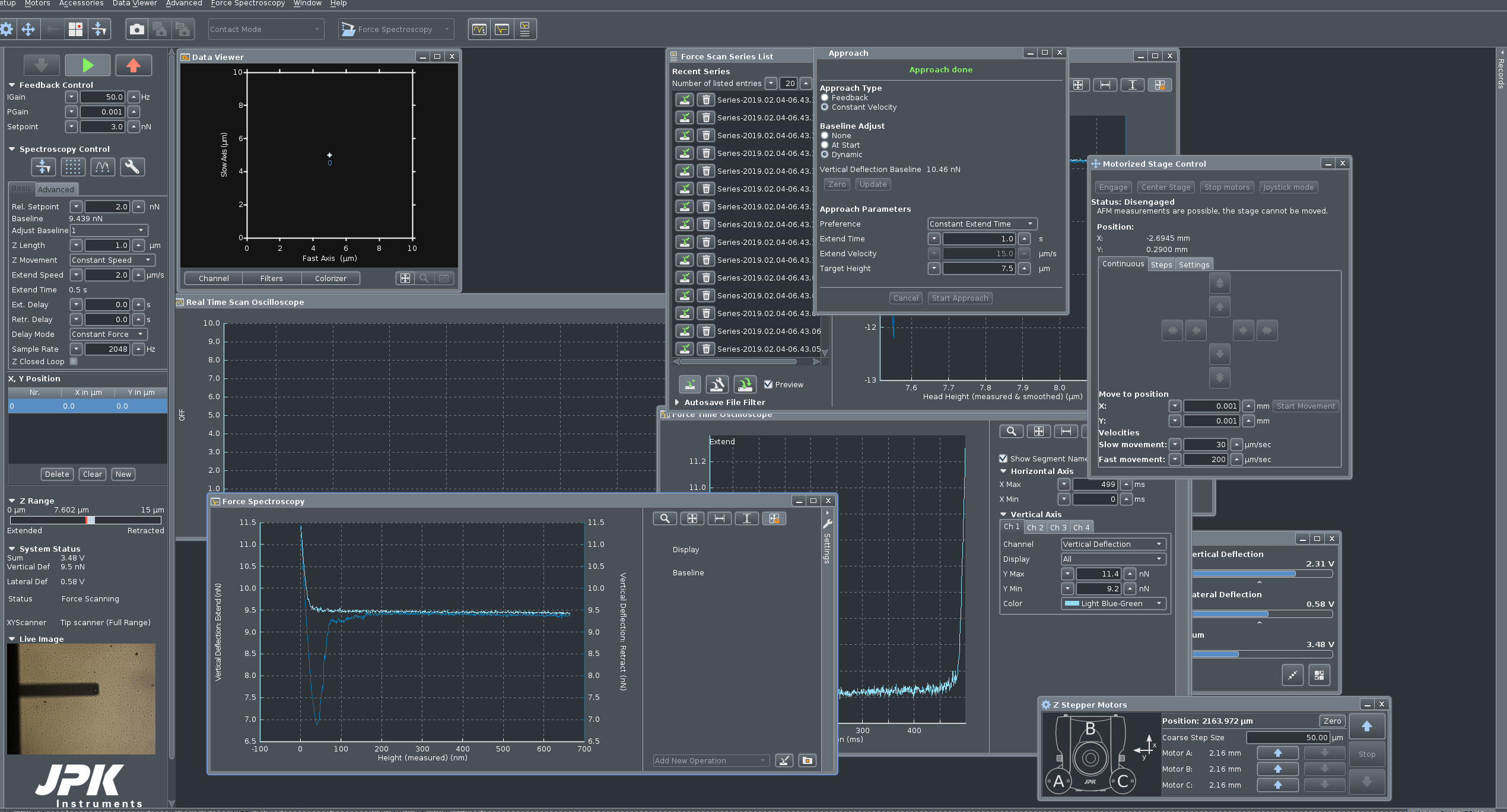

Supplement: Figure 5—source data 1. [file elife-76164-fig5-data1.zip › Figure 5 source data/System overview/Screenshot - 09.png]

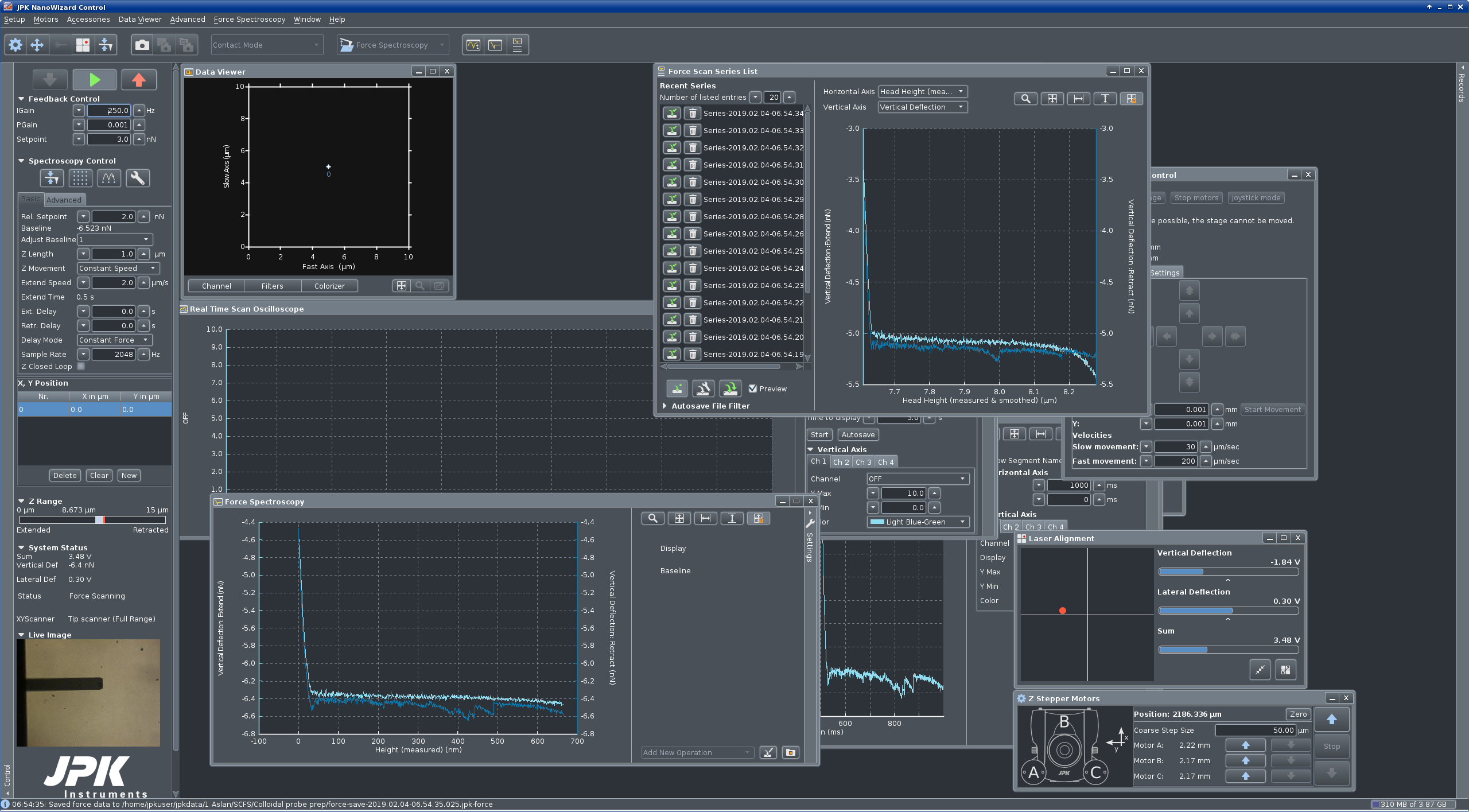

Supplement: Figure 5—source data 1. [file elife-76164-fig5-data1.zip › Figure 5 source data/System overview/Screenshot - 10.png]

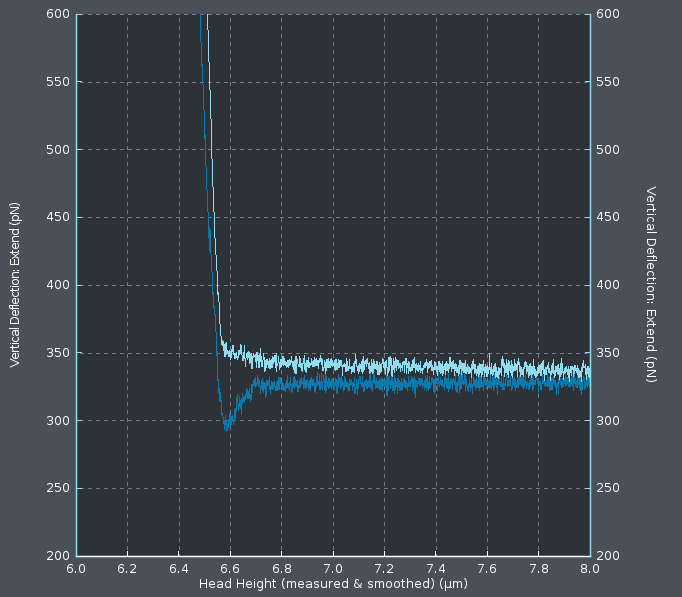

Supplement: Figure 6—source data 1. [file elife-76164-fig6-data1.zip › Figure 6 source data/FS ss/30-50um glass beads.tif]

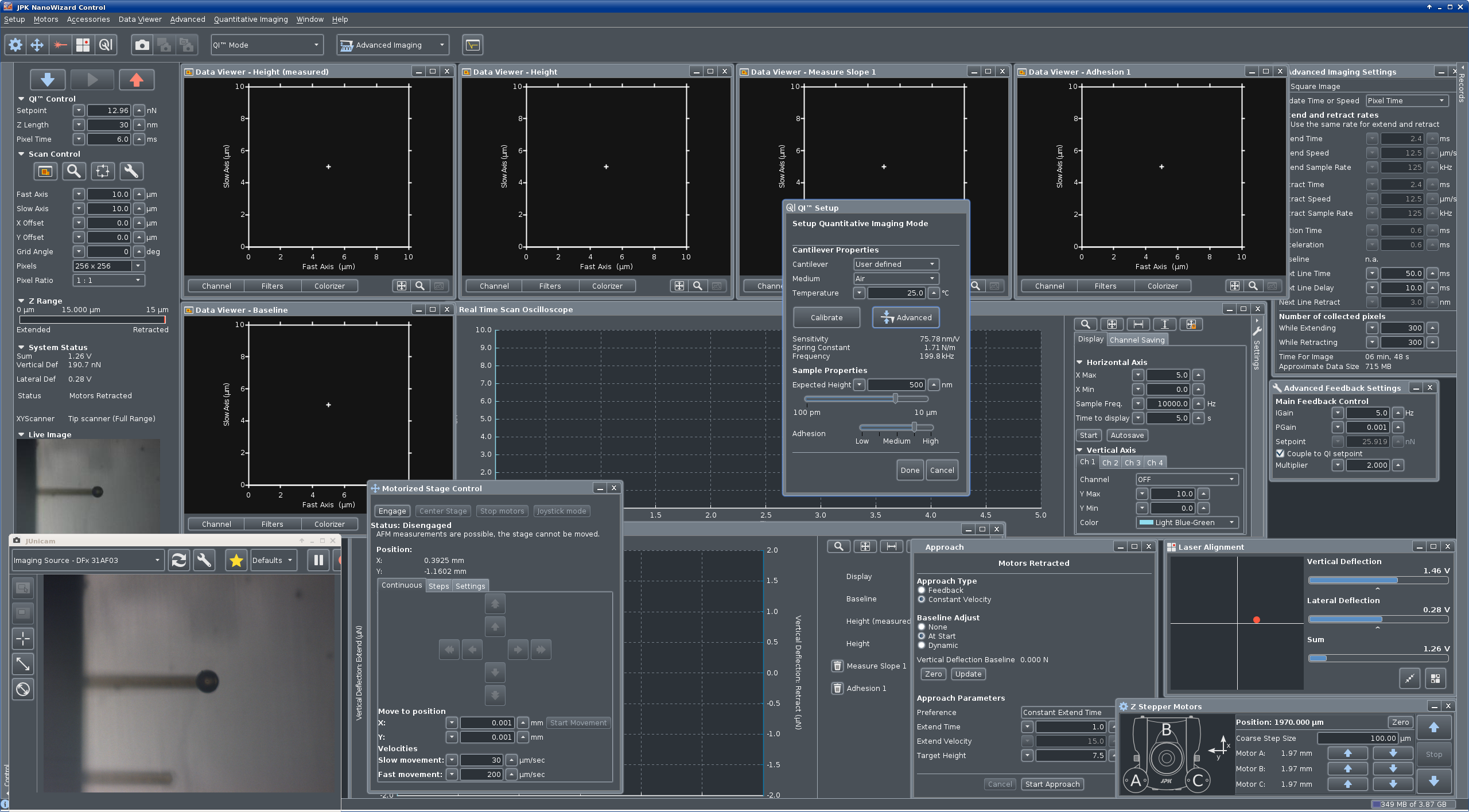

Supplement: Figure 6—source data 1. [file elife-76164-fig6-data1.zip › Figure 6 source data/FS ss/Screenshot - 09172018 - 09_28_35 AM.png]

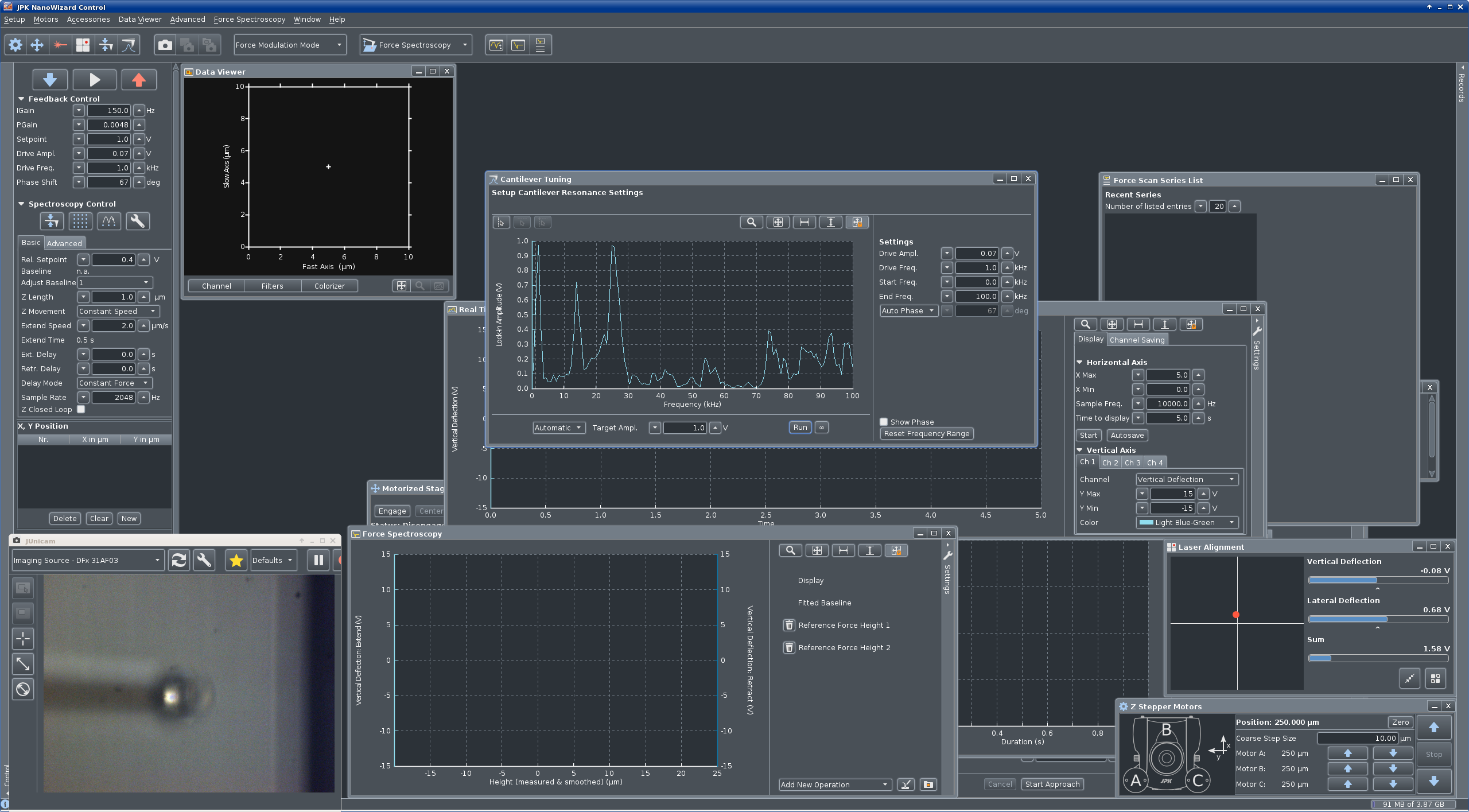

Supplement: Figure 6—source data 1. [file elife-76164-fig6-data1.zip › Figure 6 source data/FS ss/Screenshot - 09262018 - 11_08_58 PM.png]

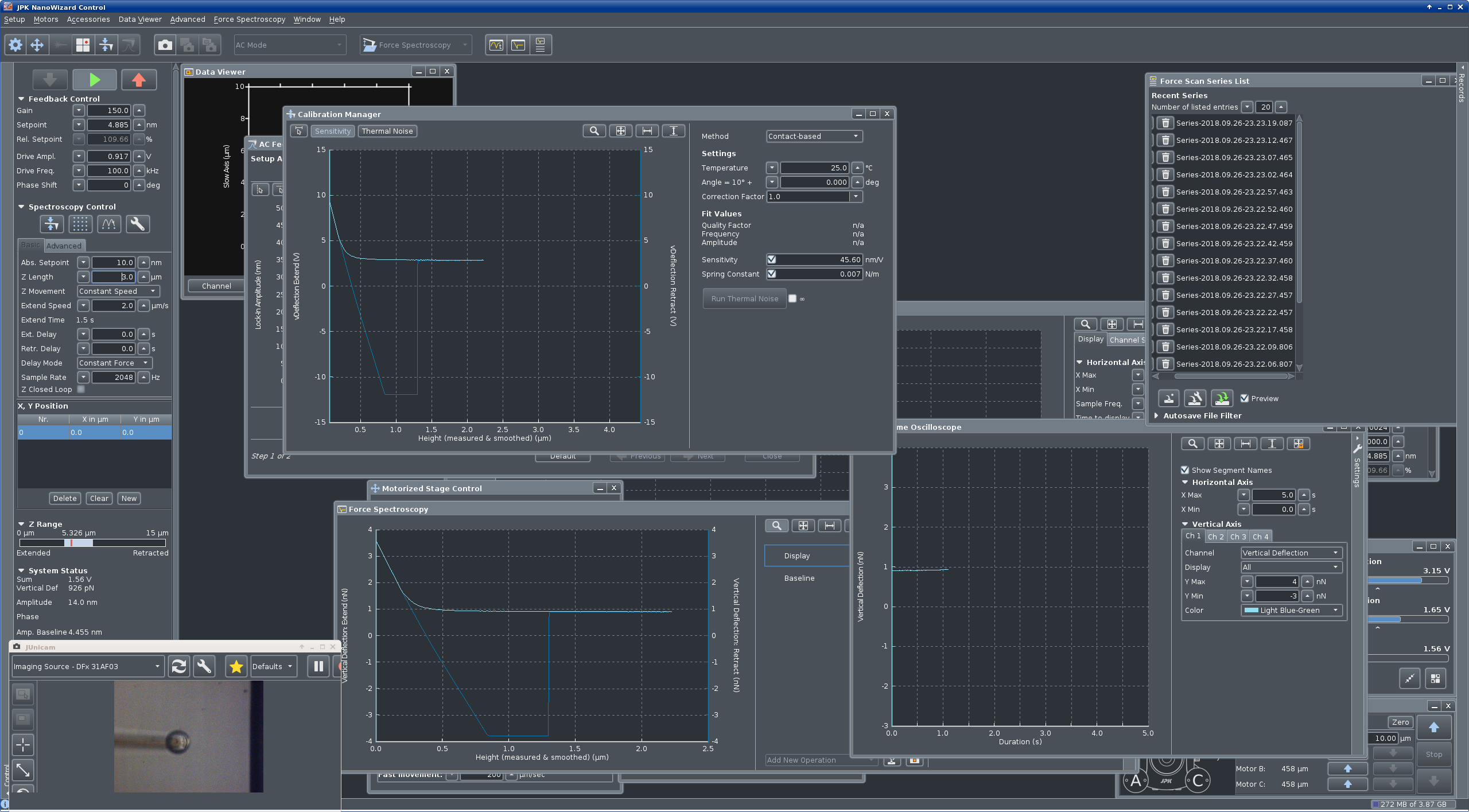

Supplement: Figure 6—source data 1. [file elife-76164-fig6-data1.zip › Figure 6 source data/FS ss/Screenshot - 09262018 - 11_23_23 PM.png]

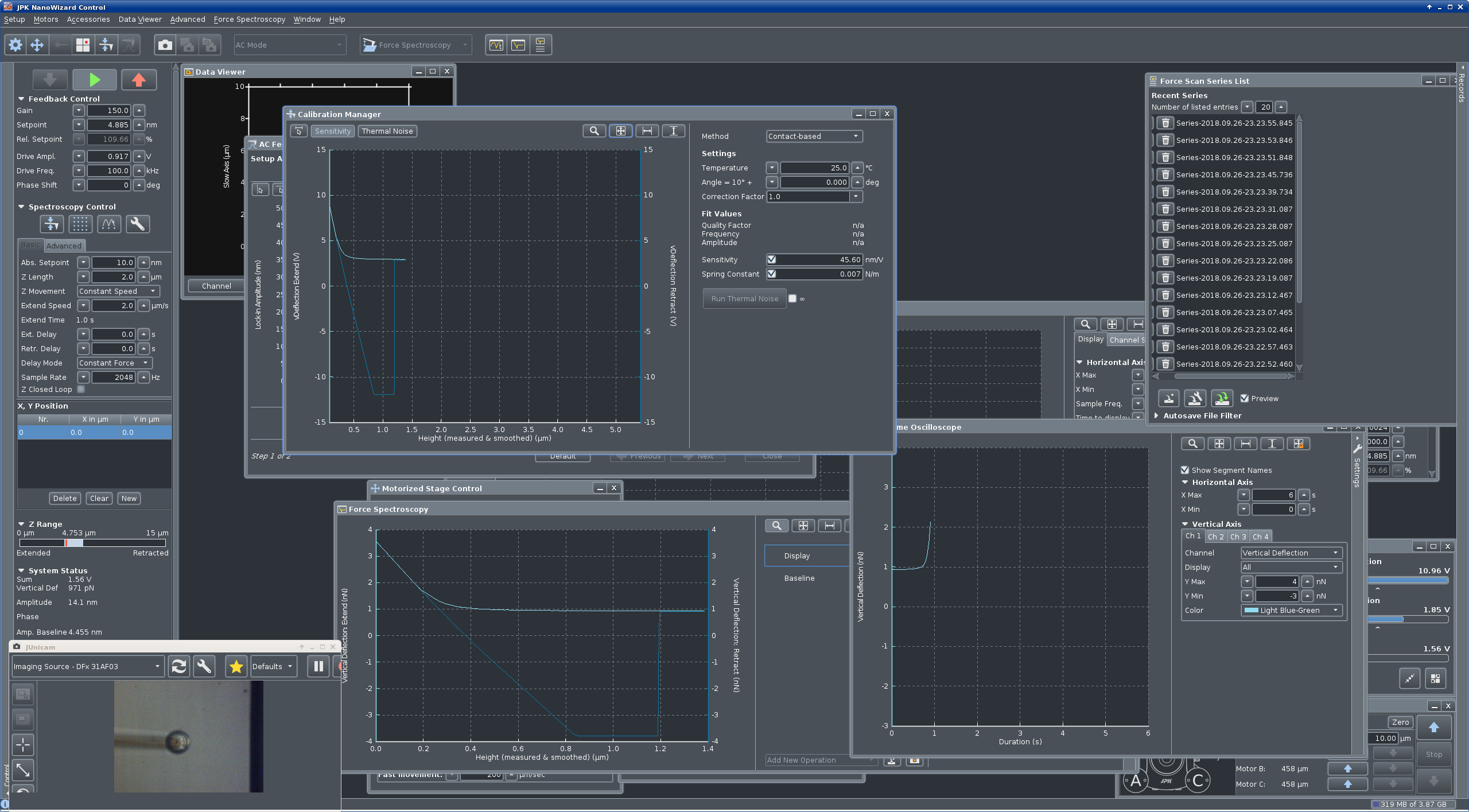

Supplement: Figure 6—source data 1. [file elife-76164-fig6-data1.zip › Figure 6 source data/FS ss/Screenshot - 09262018 - 11_23_59 PM.png]

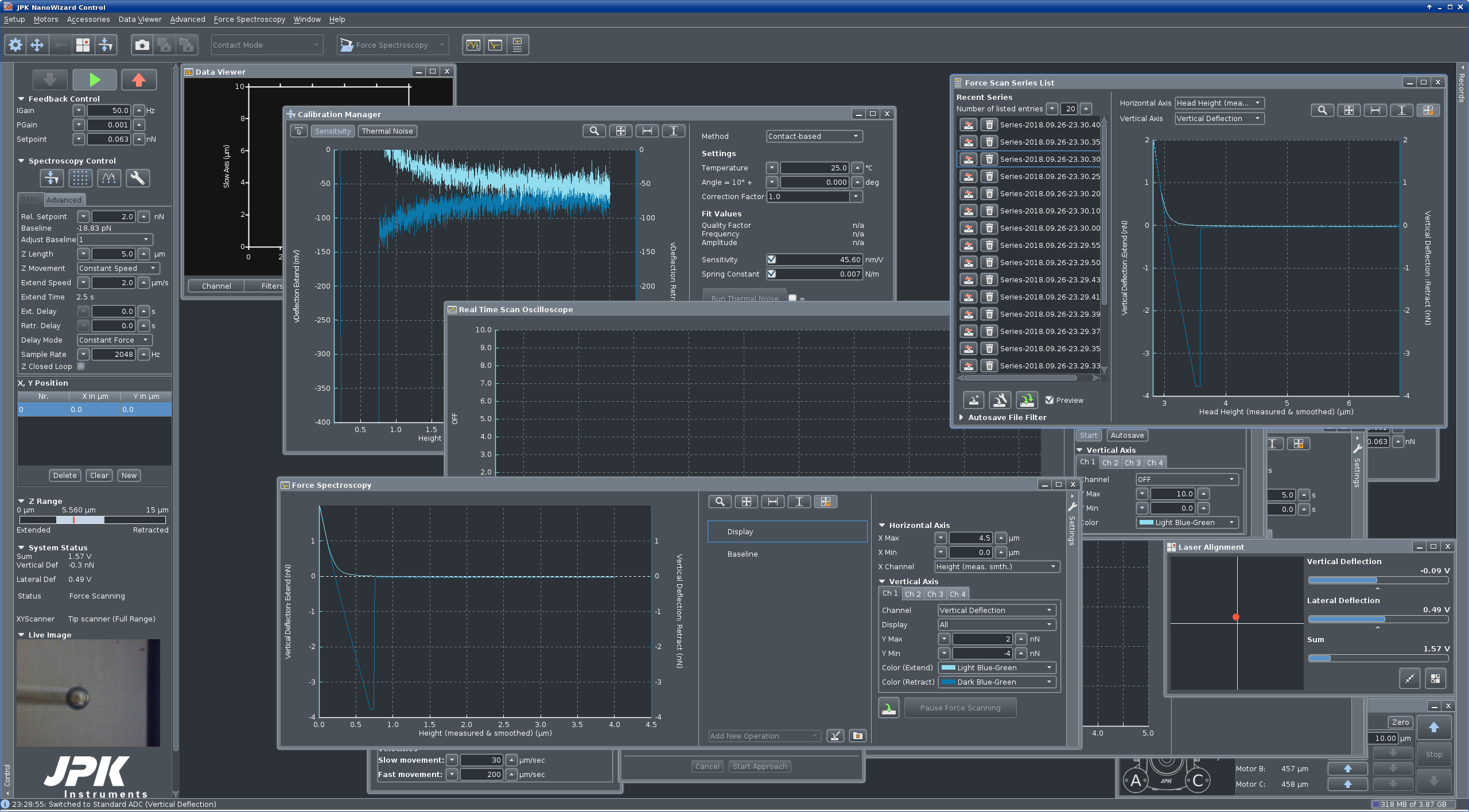

Supplement: Figure 6—source data 1. [file elife-76164-fig6-data1.zip › Figure 6 source data/FS ss/Screenshot - 09262018 - 11_30_48 PM.png]

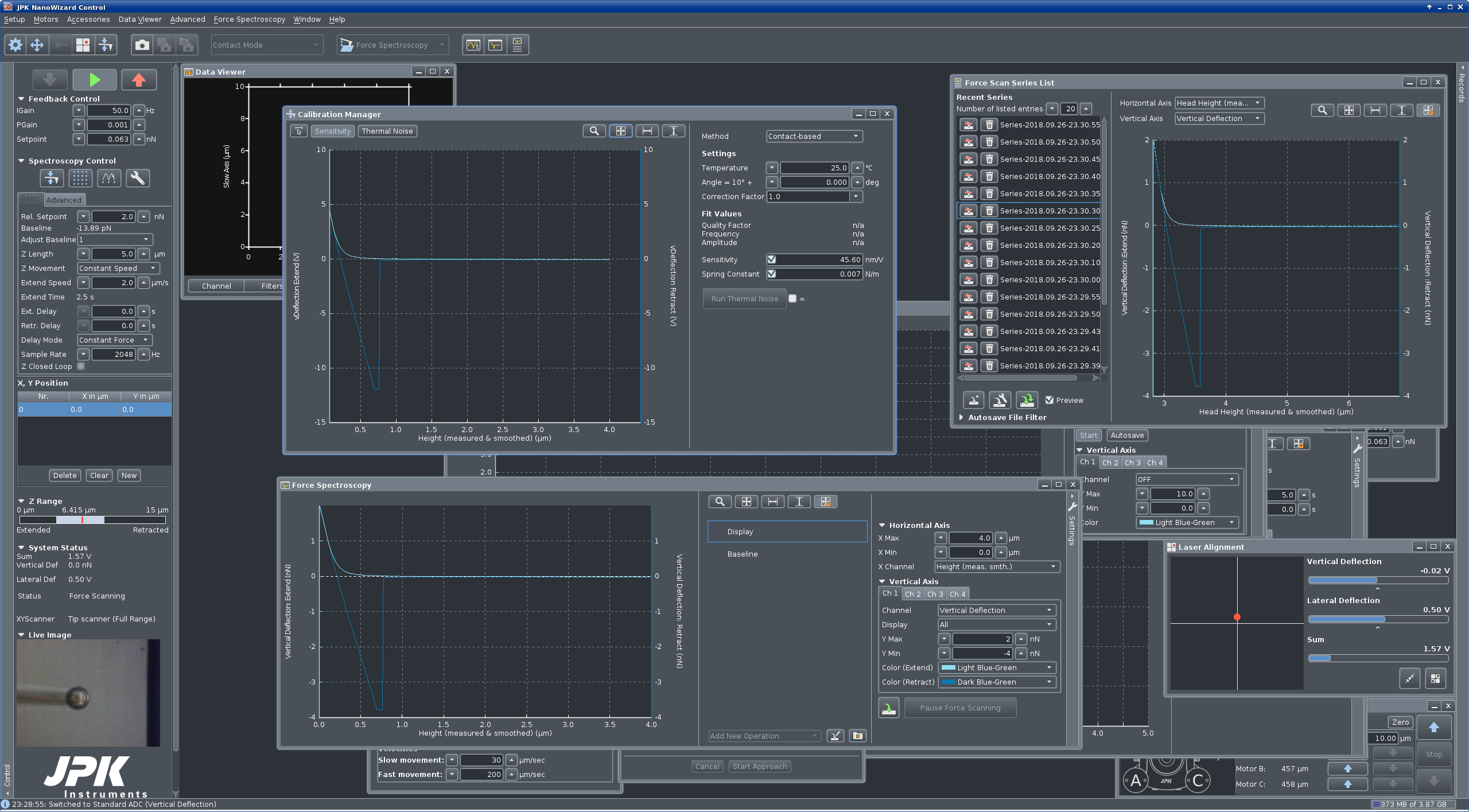

Supplement: Figure 6—source data 1. [file elife-76164-fig6-data1.zip › Figure 6 source data/FS ss/Screenshot - 09262018 - 11_31_00 PM.png]

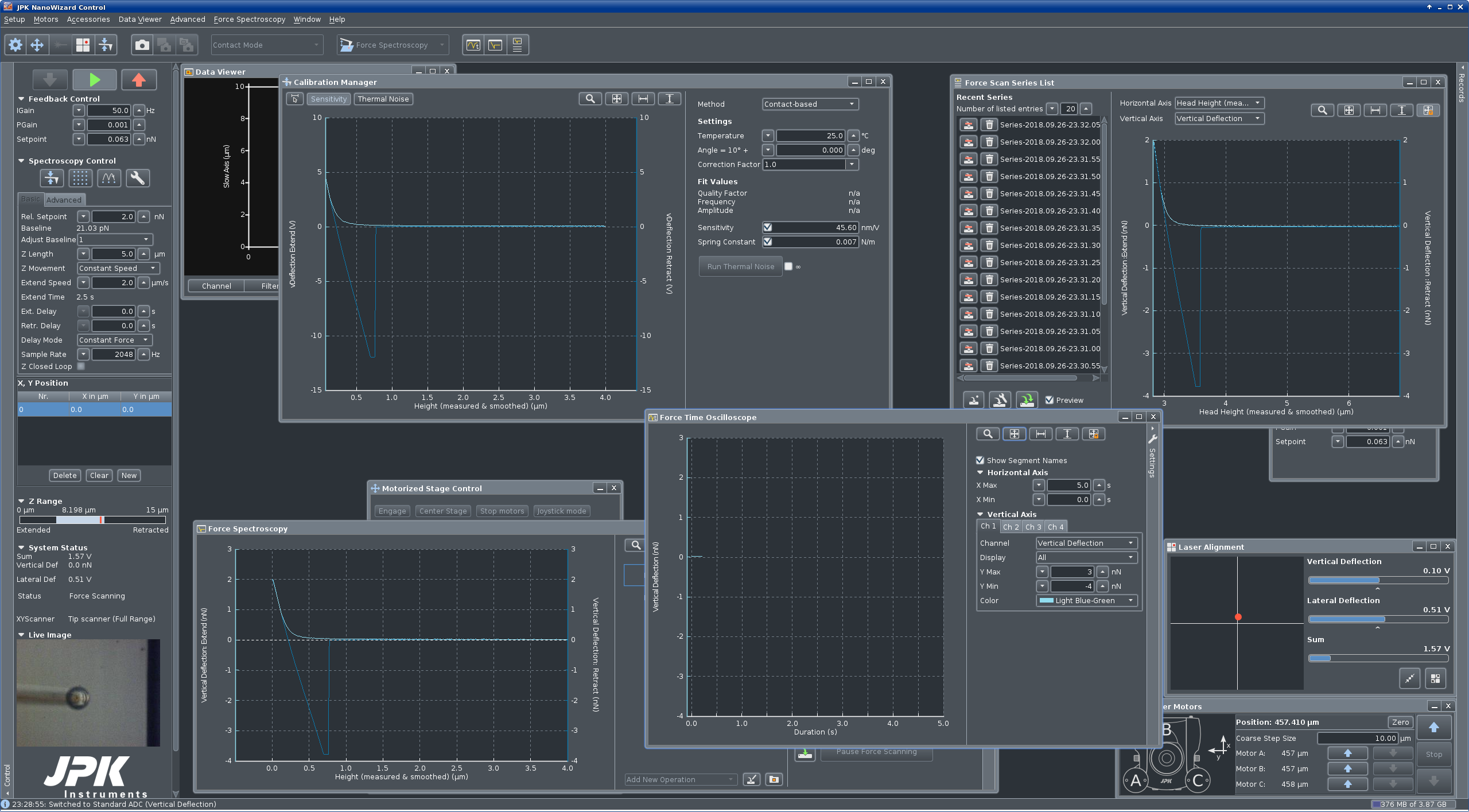

Supplement: Figure 6—source data 1. [file elife-76164-fig6-data1.zip › Figure 6 source data/FS ss/Screenshot - 09262018 - 11_32_11 PM.png]

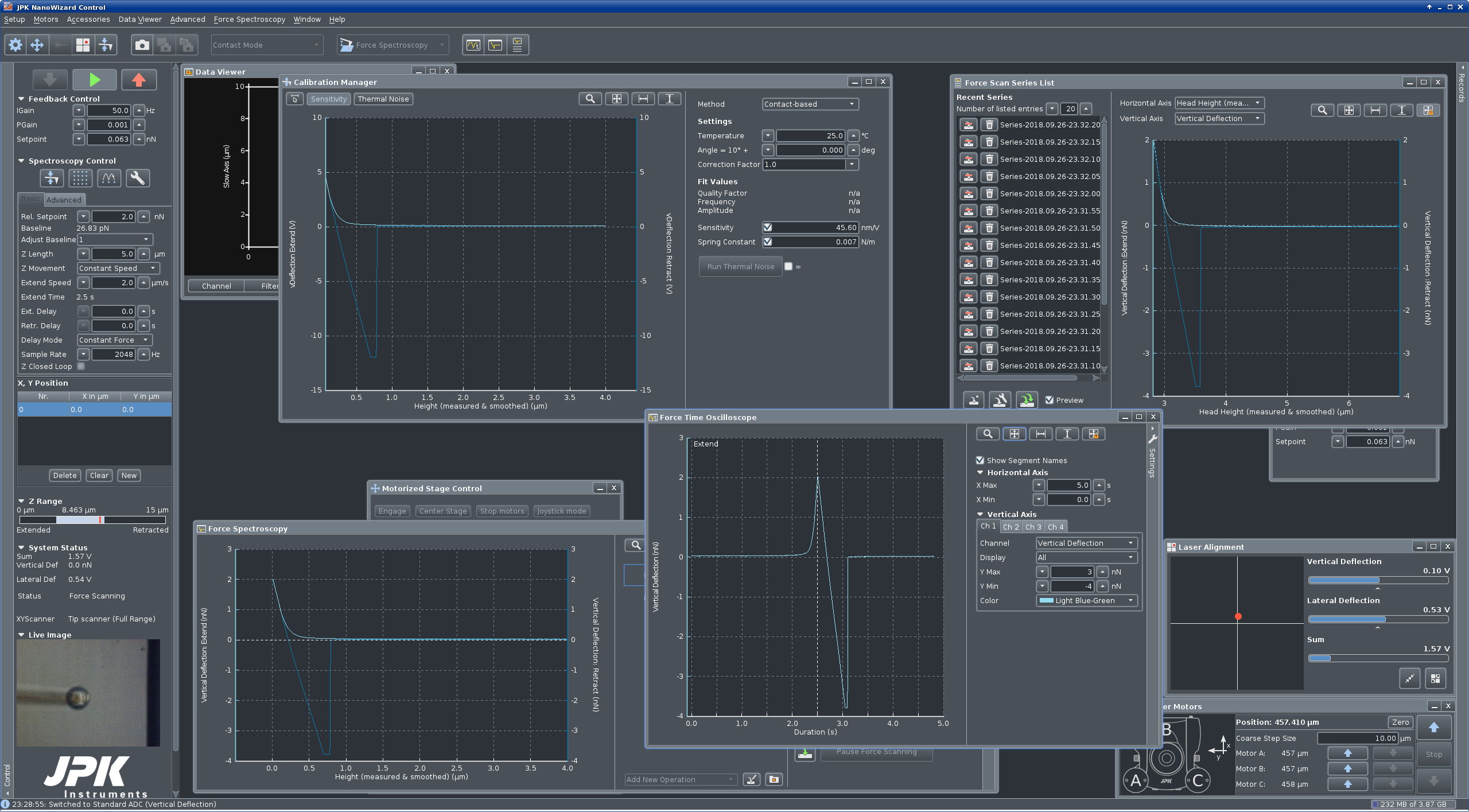

Supplement: Figure 6—source data 1. [file elife-76164-fig6-data1.zip › Figure 6 source data/FS ss/Screenshot - 09262018 - 11_32_30 PM.png]

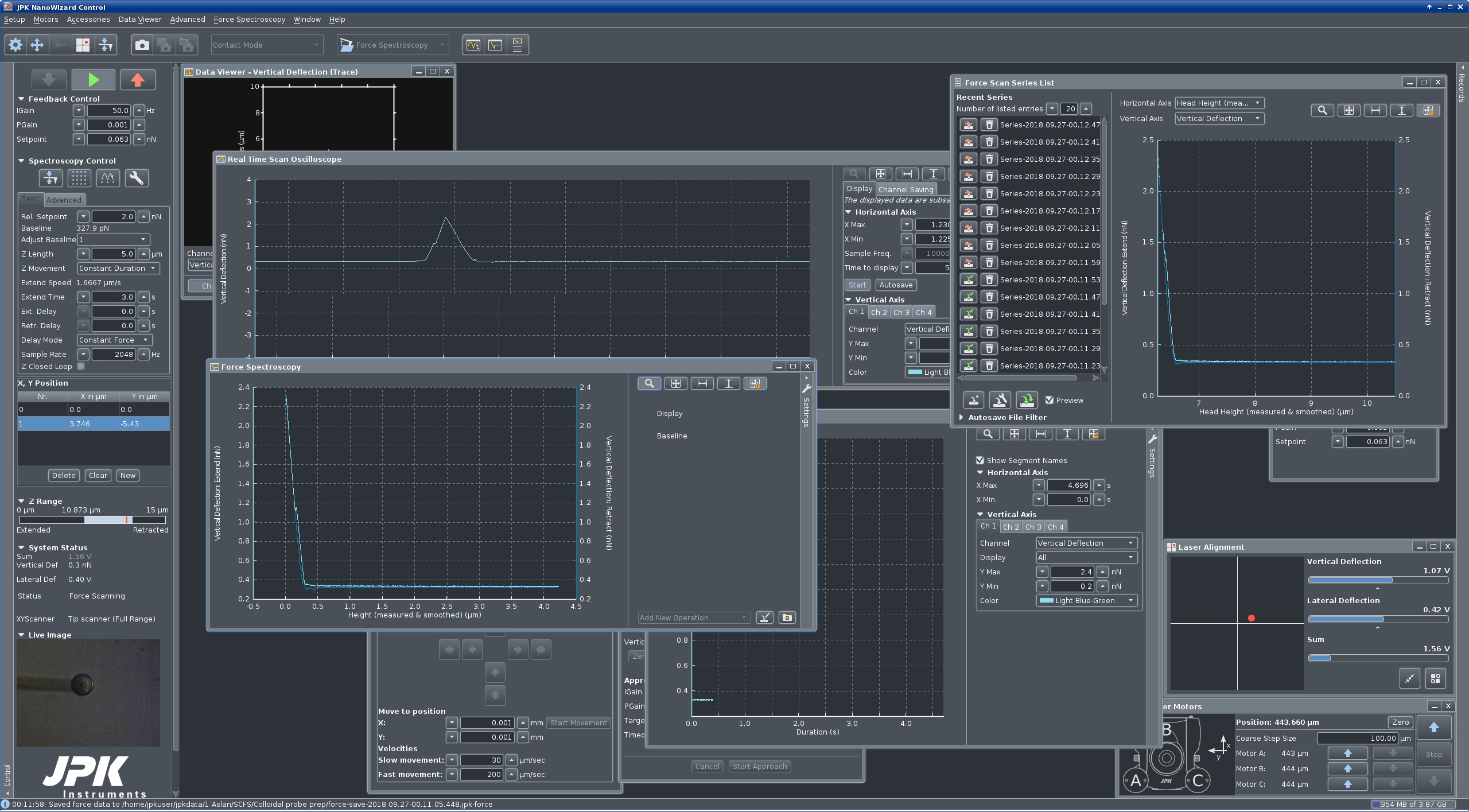

Supplement: Figure 6—source data 1. [file elife-76164-fig6-data1.zip › Figure 6 source data/FS ss/Screenshot - 09272018 - 12_12_54 AM.png]

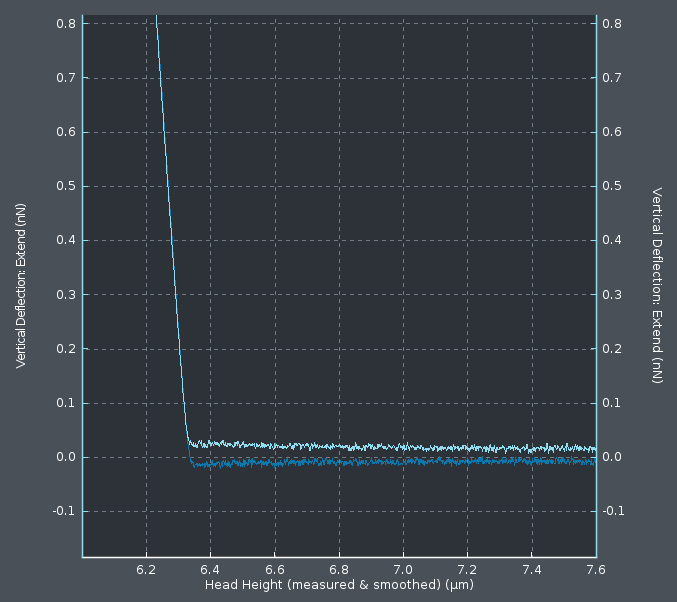

Supplement: Figure 6—source data 1. [file elife-76164-fig6-data1.zip › Figure 6 source data/FS ss/Screenshot - 09272018 - 12_20_55 AM.png]

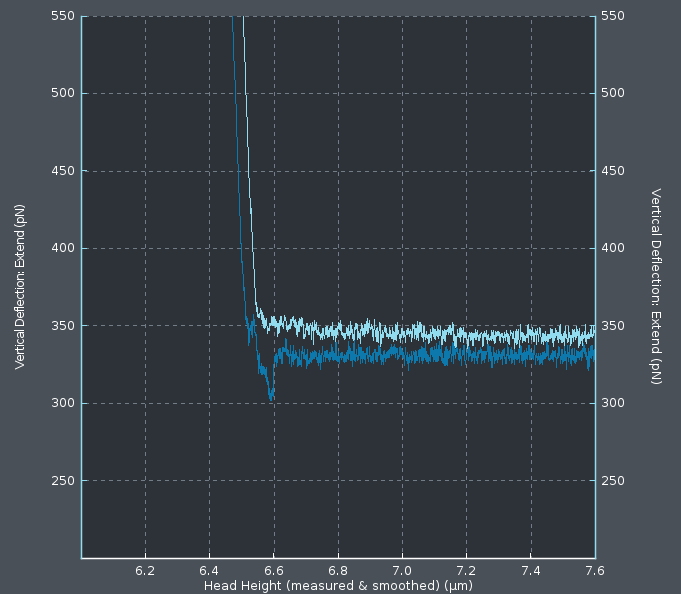

Supplement: Figure 6—source data 1. [file elife-76164-fig6-data1.zip › Figure 6 source data/FS ss/Screenshot - 09272018 - 12_23_04 AM.png]

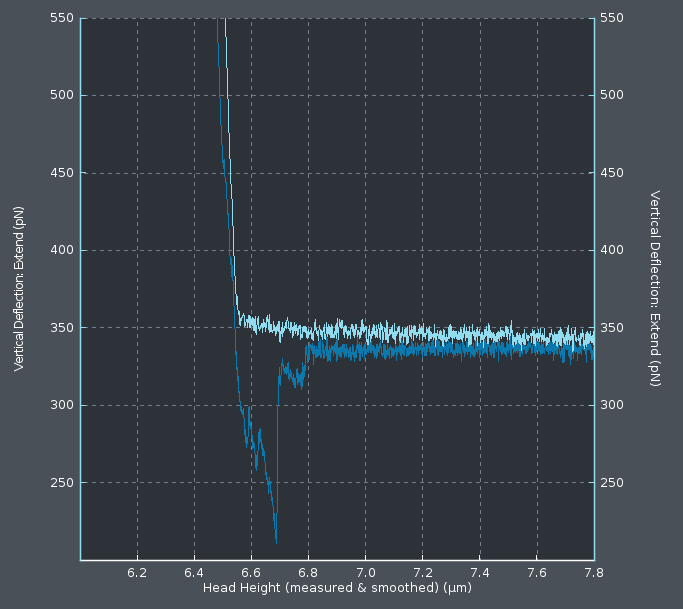

Supplement: Figure 6—source data 1. [file elife-76164-fig6-data1.zip › Figure 6 source data/FS ss/Screenshot - 09272018 - 12_23_42 AM.png]

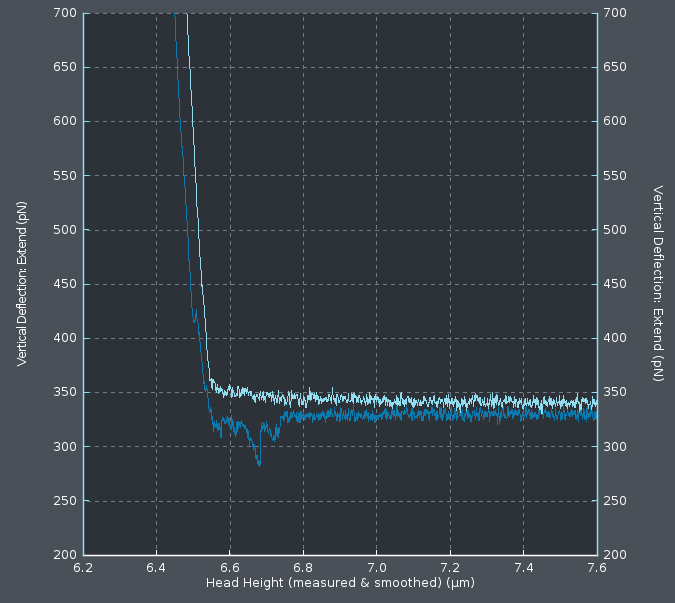

Supplement: Figure 6—source data 1. [file elife-76164-fig6-data1.zip › Figure 6 source data/FS ss/Screenshot - 09272018 - 12_24_22 AM.png]

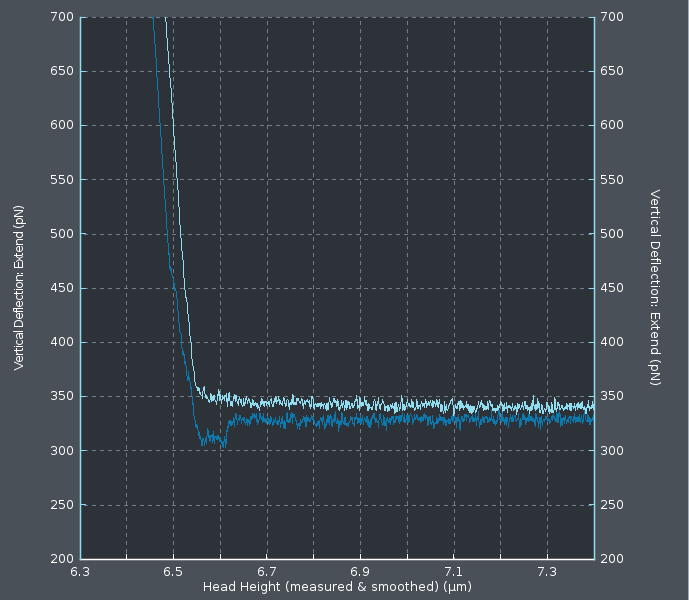

Supplement: Figure 6—source data 1. [file elife-76164-fig6-data1.zip › Figure 6 source data/FS ss/Screenshot - 09272018 - 12_24_49 AM.png]

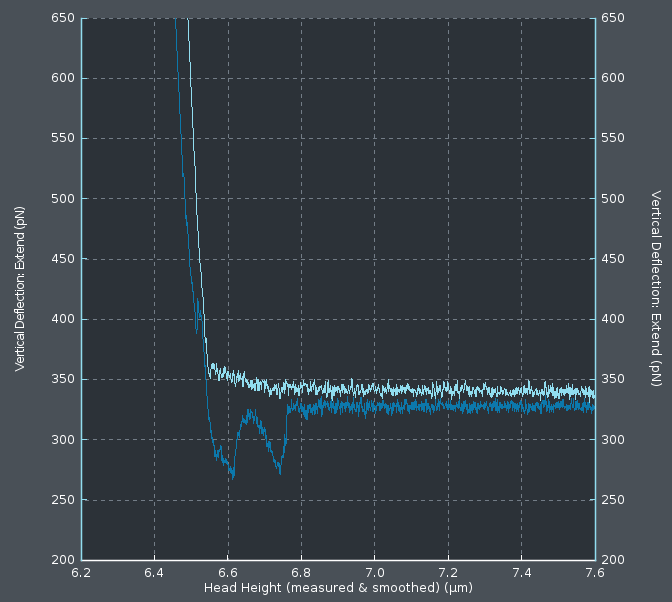

Supplement: Figure 6—source data 1. [file elife-76164-fig6-data1.zip › Figure 6 source data/FS ss/Screenshot - 09272018 - 12_25_45 AM.png]

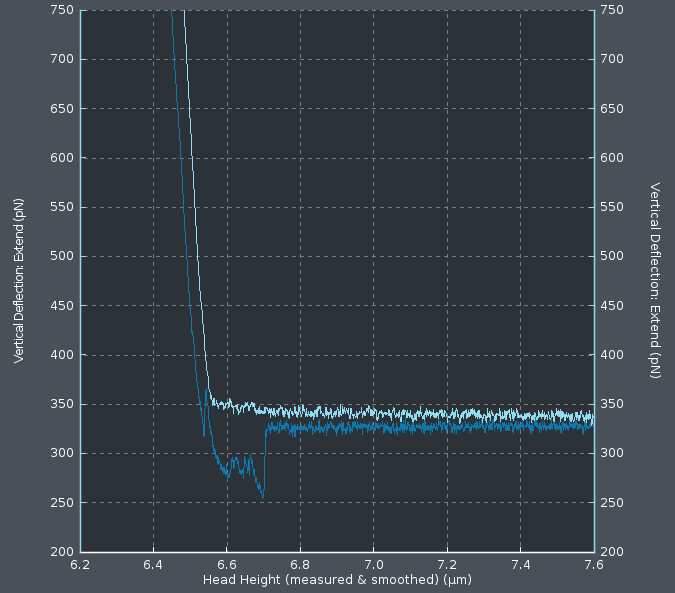

Supplement: Figure 6—source data 1. [file elife-76164-fig6-data1.zip › Figure 6 source data/FS ss/Screenshot - 09272018 - 12_26_23 AM.png]

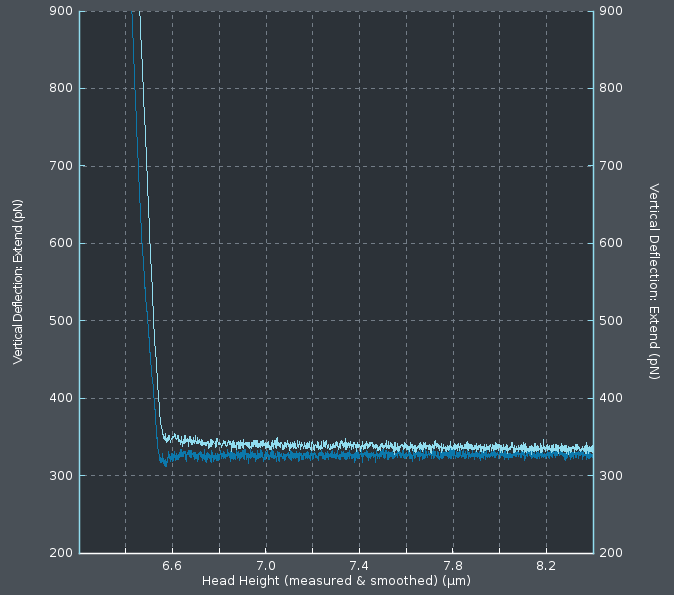

Supplement: Figure 6—source data 1. [file elife-76164-fig6-data1.zip › Figure 6 source data/FS ss/Screenshot - 09272018 - 12_26_56 AM.png]

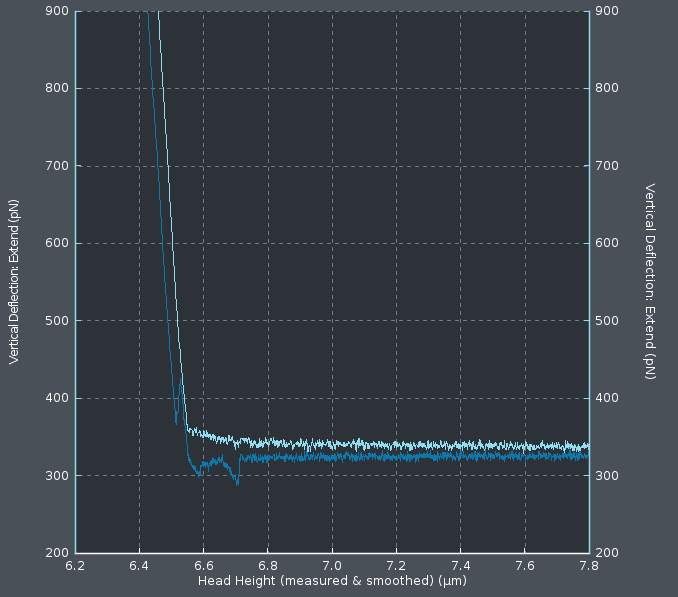

Supplement: Figure 6—source data 1. [file elife-76164-fig6-data1.zip › Figure 6 source data/FS ss/Screenshot - 09272018 - 12_27_27 AM.png]

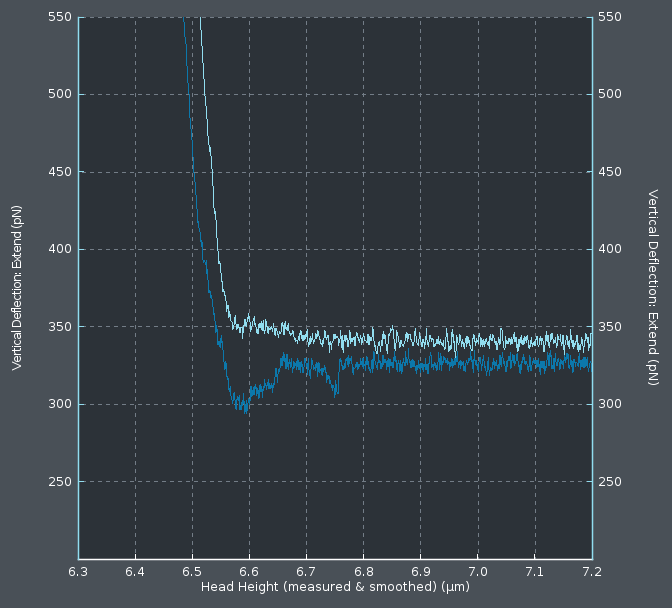

Supplement: Figure 6—source data 1. [file elife-76164-fig6-data1.zip › Figure 6 source data/FS ss/Screenshot - 09272018 - 12_27_58 AM.png]

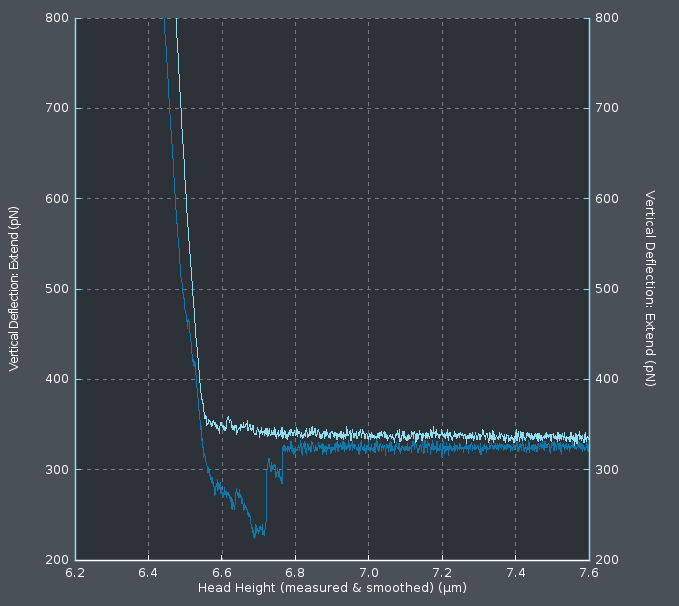

Supplement: Figure 6—source data 1. [file elife-76164-fig6-data1.zip › Figure 6 source data/FS ss/Screenshot - 09272018 - 12_28_46 AM.png]

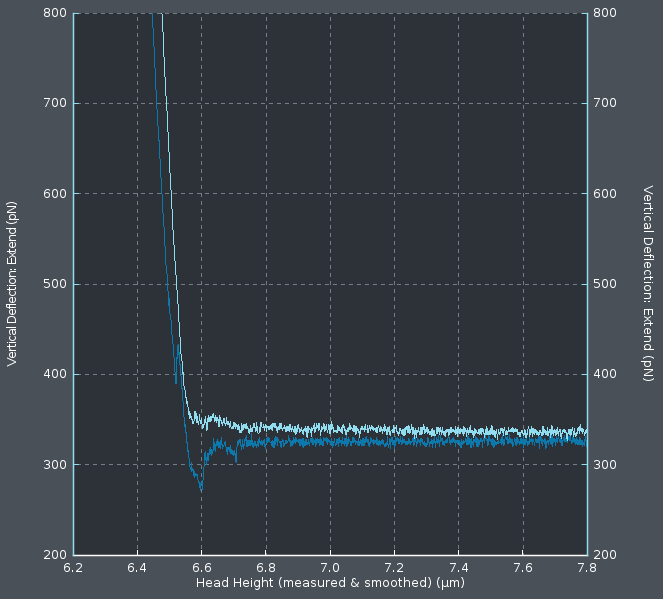

Supplement: Figure 6—source data 1. [file elife-76164-fig6-data1.zip › Figure 6 source data/FS ss/Screenshot - 09272018 - 12_29_43 AM.png]

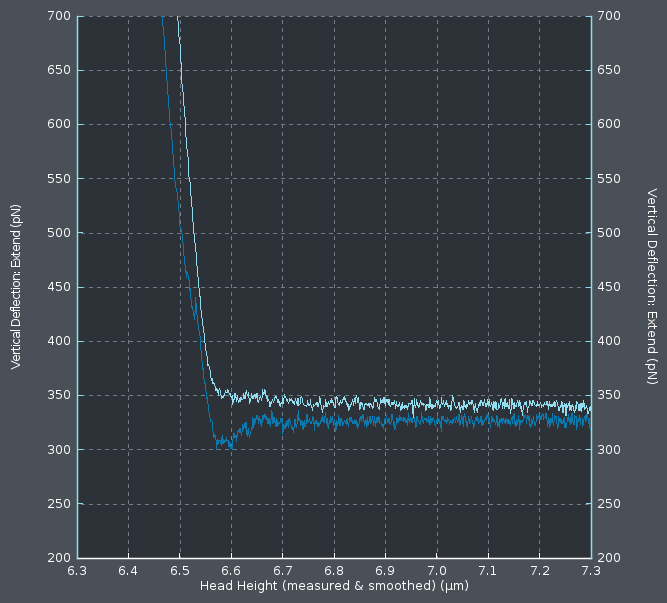

Supplement: Figure 6—source data 1. [file elife-76164-fig6-data1.zip › Figure 6 source data/FS ss/Screenshot - 09272018 - 12_30_15 AM.png]

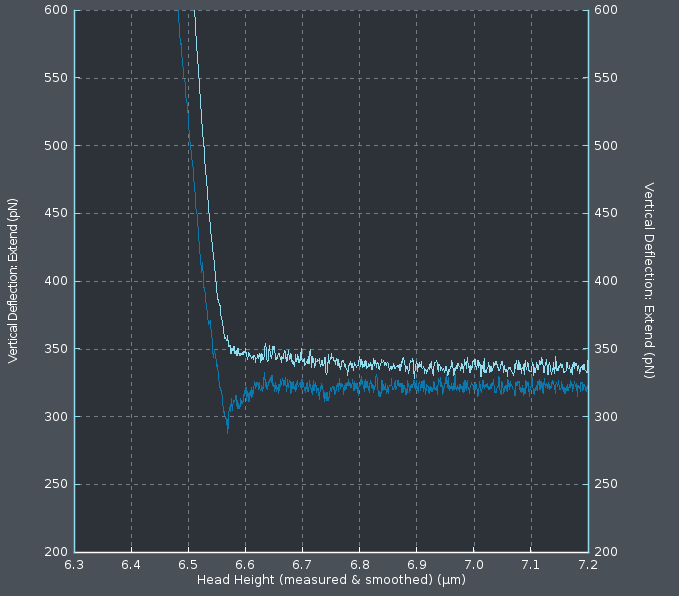

Supplement: Figure 6—source data 1. [file elife-76164-fig6-data1.zip › Figure 6 source data/FS ss/Screenshot - 09272018 - 12_30_46 AM.png]

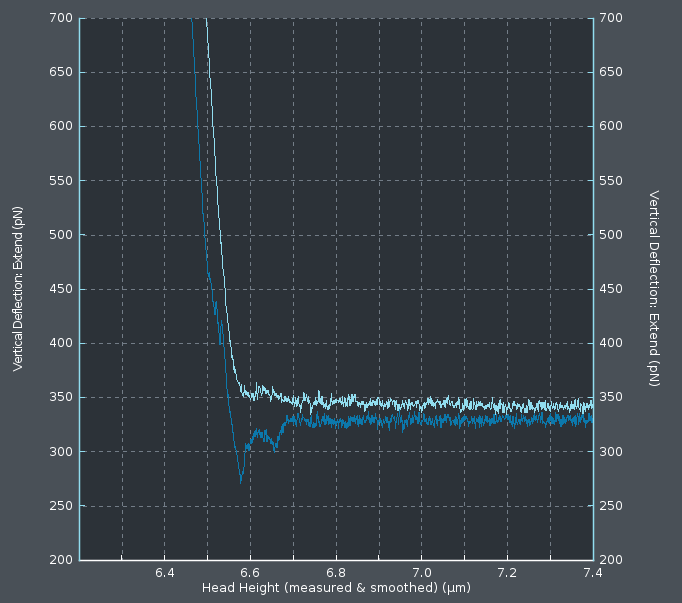

Supplement: Figure 6—source data 1. [file elife-76164-fig6-data1.zip › Figure 6 source data/FS ss/Screenshot - 09272018 - 12_31_16 AM.png]

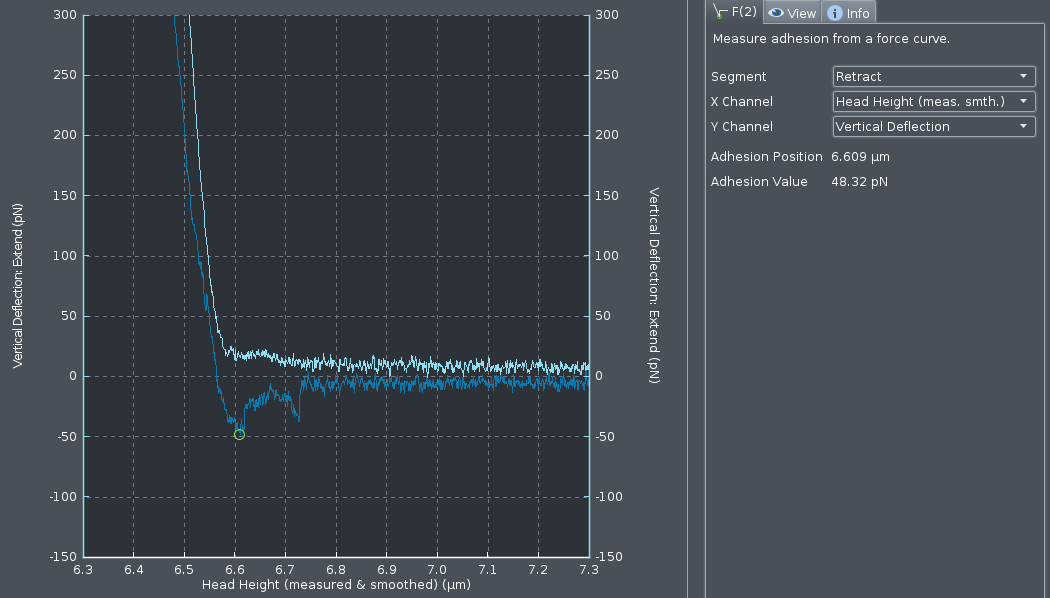

Supplement: Figure 6—source data 1. [file elife-76164-fig6-data1.zip › Figure 6 source data/FS ss/Screenshot - 09272018 - 12_32_27 AM.png]

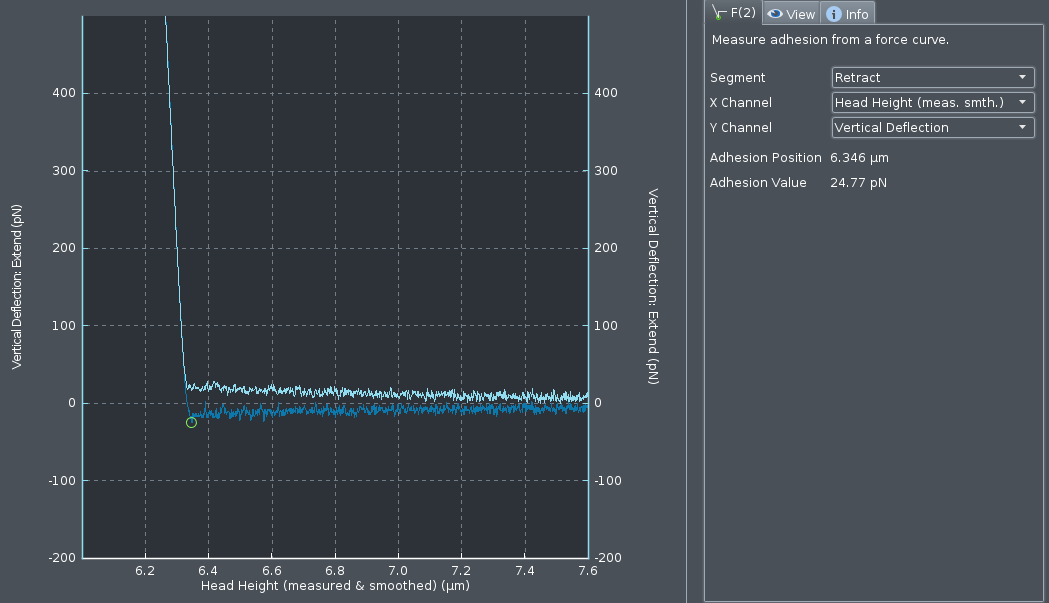

Supplement: Figure 6—source data 1. [file elife-76164-fig6-data1.zip › Figure 6 source data/FS ss/Screenshot - 09272018 - 12_34_06 AM.png]

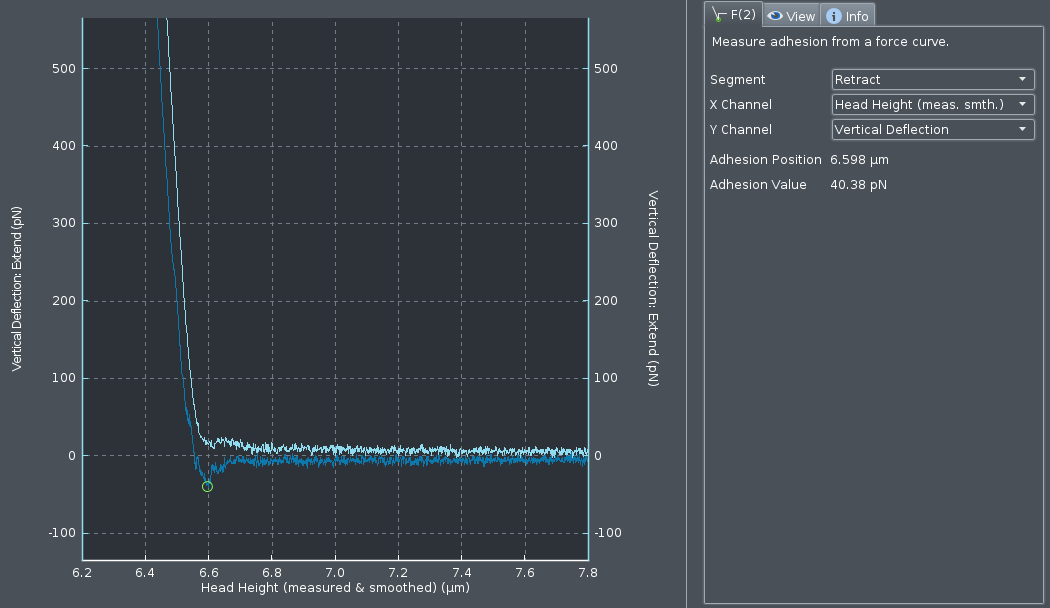

Supplement: Figure 6—source data 1. [file elife-76164-fig6-data1.zip › Figure 6 source data/FS ss/Screenshot - 09272018 - 12_34_24 AM.png]

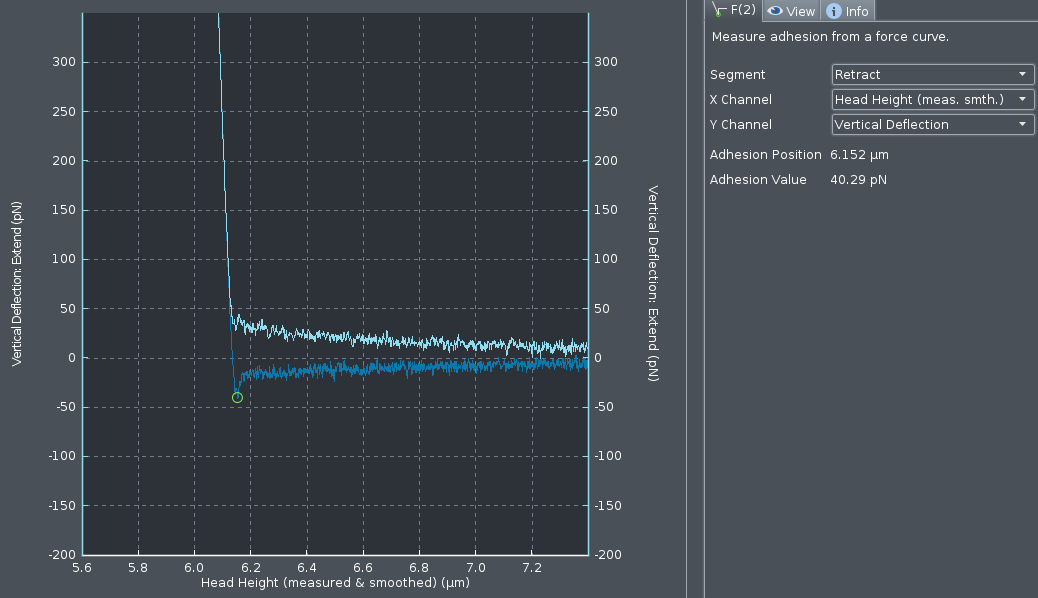

Supplement: Figure 6—source data 1. [file elife-76164-fig6-data1.zip › Figure 6 source data/FS ss/Screenshot - 09272018 - 12_35_14 AM.png]

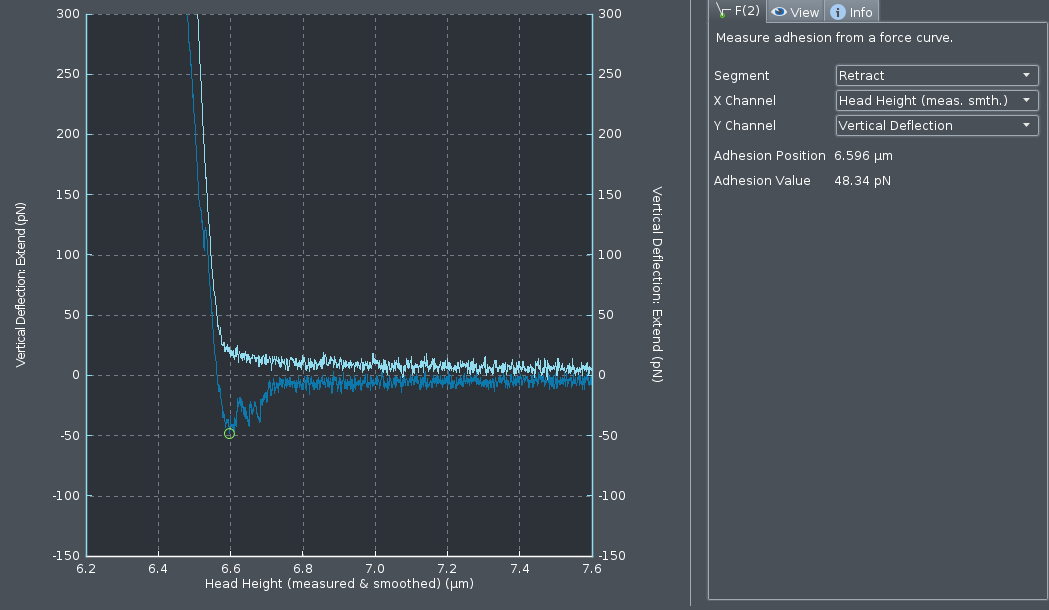

Supplement: Figure 6—source data 1. [file elife-76164-fig6-data1.zip › Figure 6 source data/FS ss/Screenshot - 09272018 - 12_35_50 AM.png]

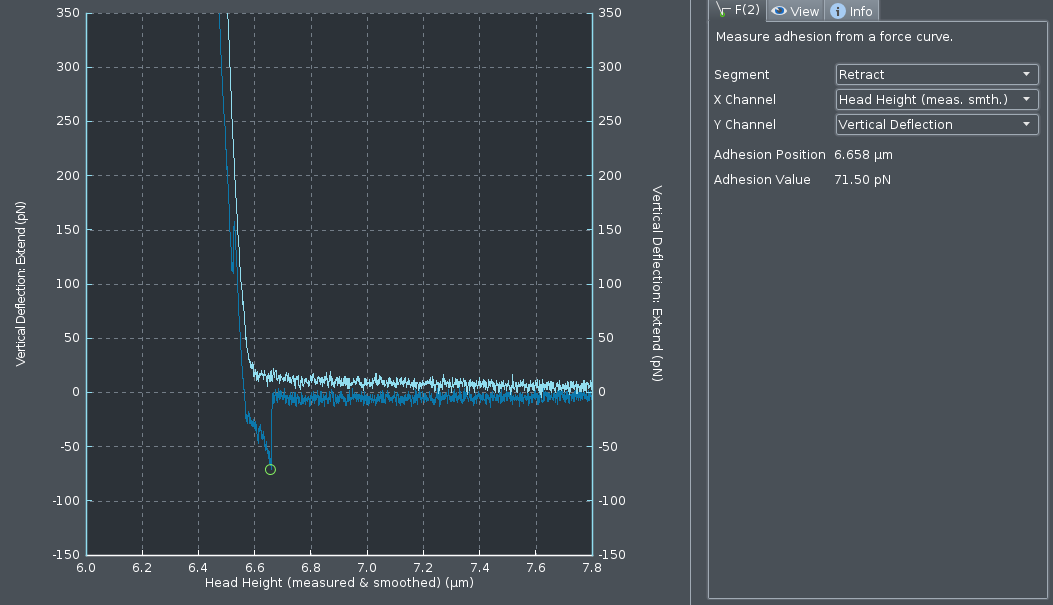

Supplement: Figure 6—source data 1. [file elife-76164-fig6-data1.zip › Figure 6 source data/FS ss/Screenshot - 09272018 - 12_37_32 AM.png]

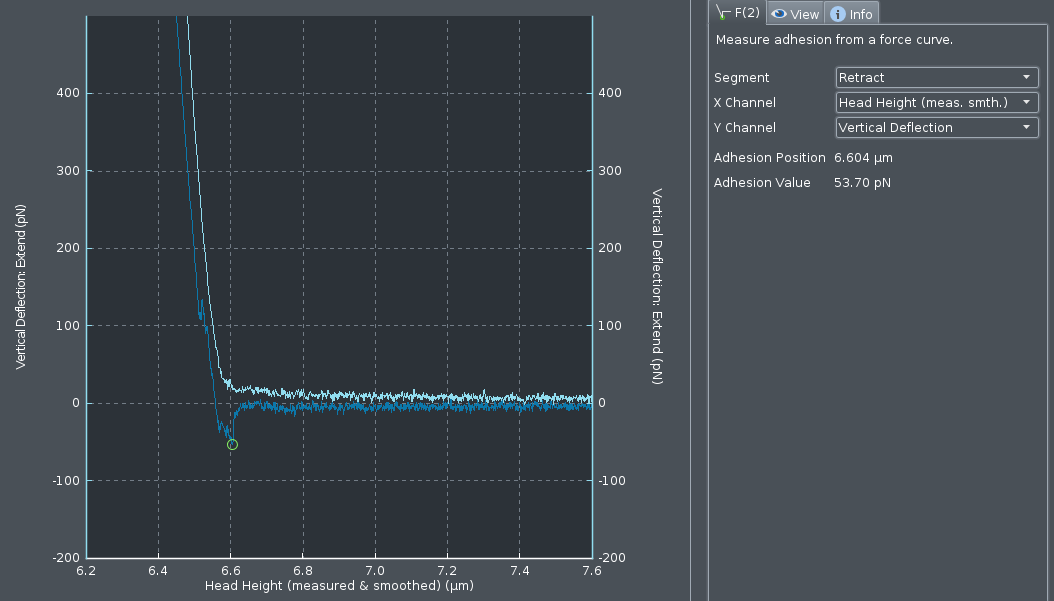

Supplement: Figure 6—source data 1. [file elife-76164-fig6-data1.zip › Figure 6 source data/FS ss/Screenshot - 09272018 - 12_38_03 AM.png]

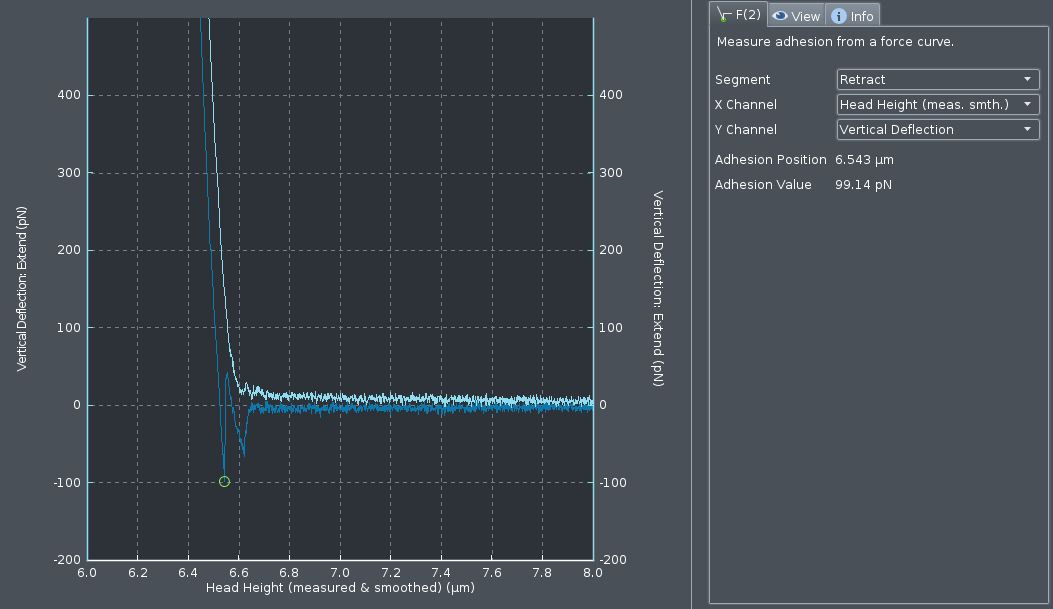

Supplement: Figure 6—source data 1. [file elife-76164-fig6-data1.zip › Figure 6 source data/FS ss/Screenshot - 09272018 - 12_38_48 AM.png]

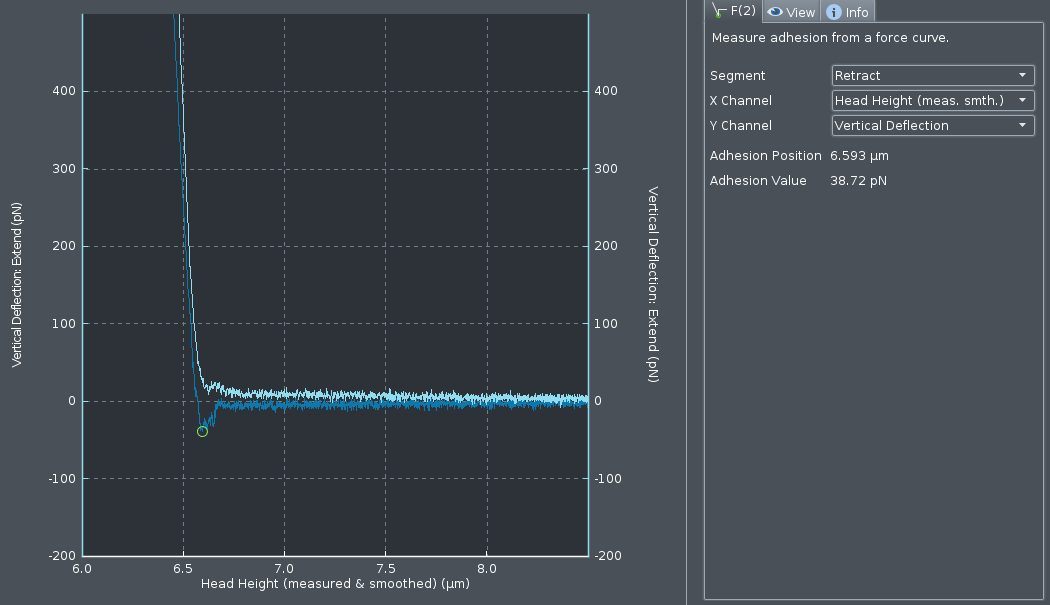

Supplement: Figure 6—source data 1. [file elife-76164-fig6-data1.zip › Figure 6 source data/FS ss/Screenshot - 09272018 - 12_39_27 AM.png]

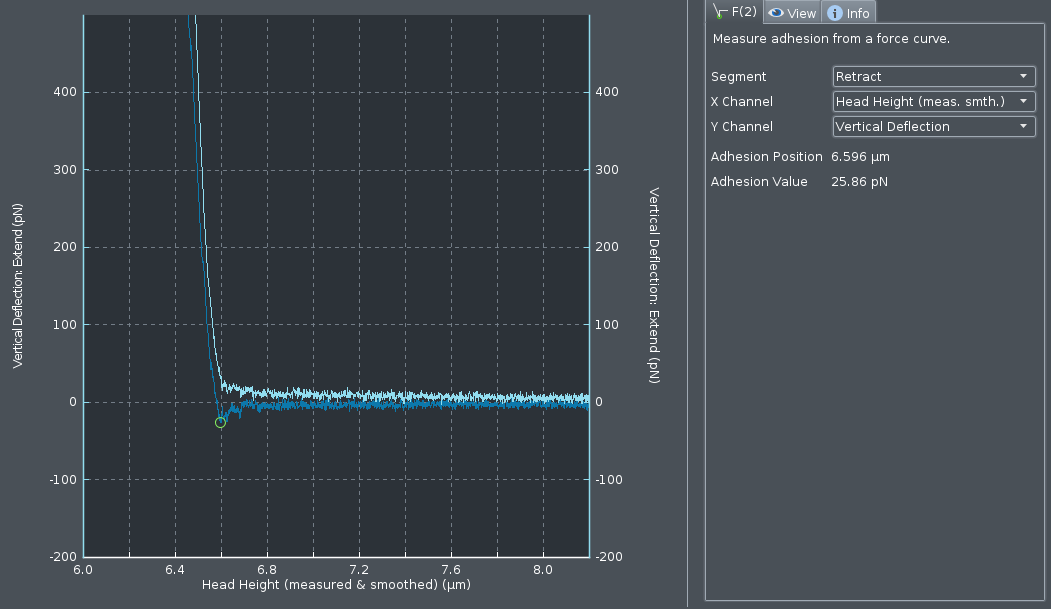

Supplement: Figure 6—source data 1. [file elife-76164-fig6-data1.zip › Figure 6 source data/FS ss/Screenshot - 09272018 - 12_40_06 AM.png]

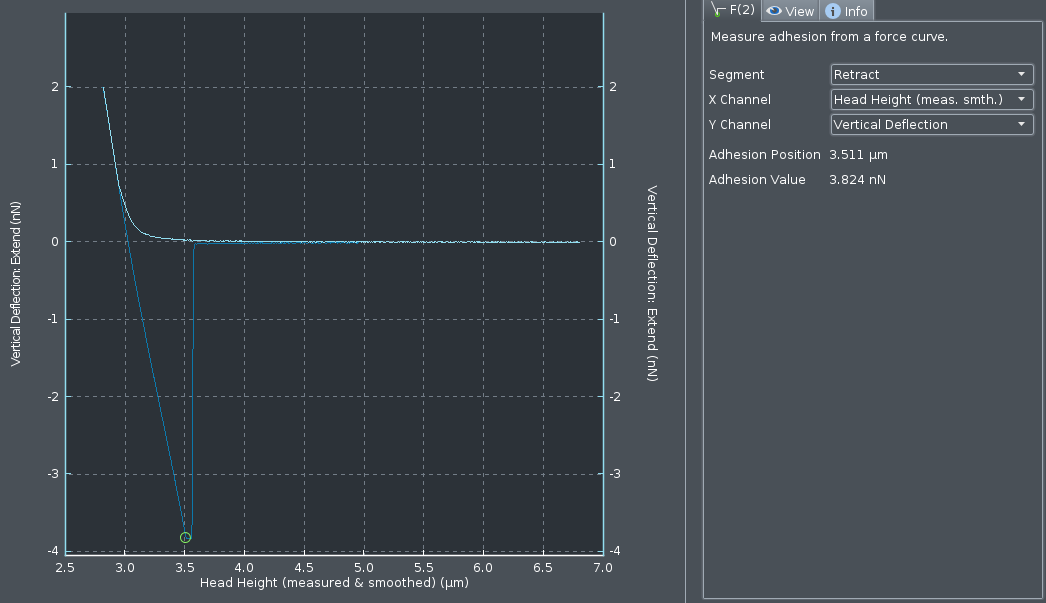

Supplement: Figure 6—source data 1. [file elife-76164-fig6-data1.zip › Figure 6 source data/FS ss/Screenshot - 09272018 - 12_43_23 AM.png]

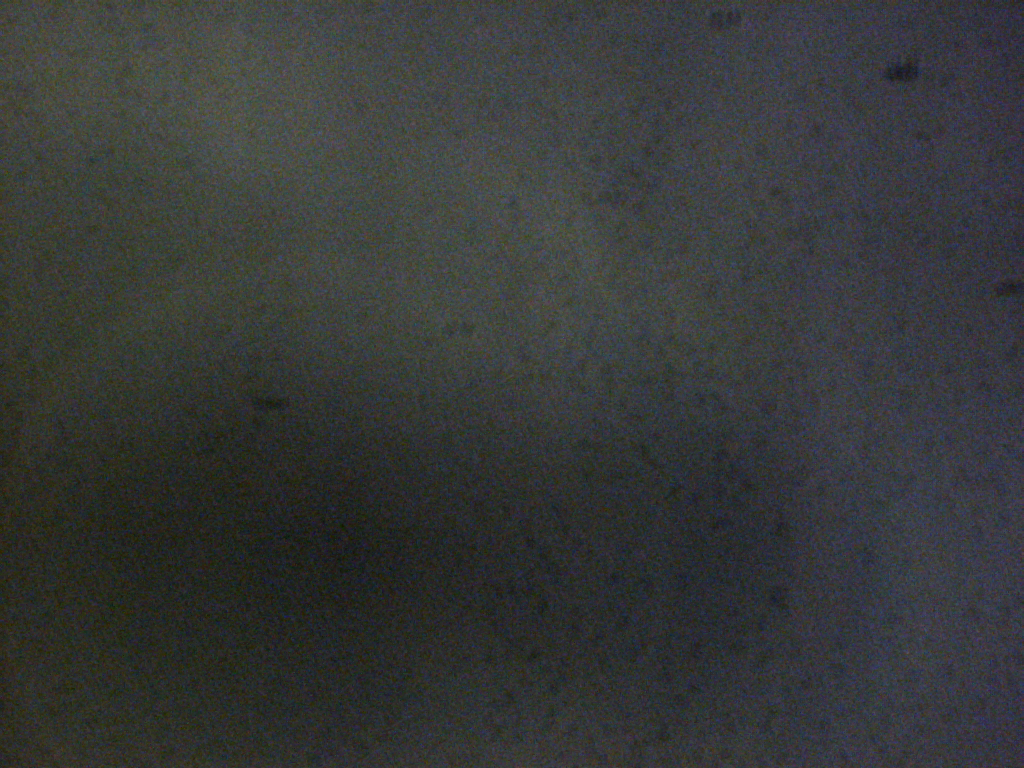

Supplement: Figure 6—source data 1. [file elife-76164-fig6-data1.zip › Figure 6 source data/FS ss/Untitled.tif]
